# Explaining empirical dynamic modelling using verbal, graphical and mathematical approaches

**DOI:** 10.1002/ece3.10903

**Published:** 2024-05-15

**Authors:** Andrew M. Edwards, Luke A. Rogers, Carrie A. Holt

**Affiliations:** ^1^ Pacific Biological Station Fisheries and Oceans Canada Nanaimo British Columbia Canada; ^2^ Department of Biology University of Victoria Victoria British Columbia Canada

**Keywords:** attractor reconstruction, delay embedding, model‐free forecasting, simplex projection, Takens' theorem

## Abstract

Empirical dynamic modelling (EDM) is becoming an increasingly popular method for understanding the dynamics of ecosystems. It has been applied to laboratory, terrestrial, freshwater and marine systems, used to forecast natural populations and has addressed fundamental ecological questions. Despite its increasing use, we have not found full explanations of EDM in the ecological literature, limiting understanding and reproducibility. Here we expand upon existing work by providing a detailed introduction to EDM. We use three progressively more complex approaches. A short verbal explanation of EDM is then explicitly demonstrated by graphically working through a simple example. We then introduce a full mathematical description of the steps involved. Conceptually, EDM translates a time series of data into a path through a multi‐dimensional space, whose axes are lagged values of the time series. A time step is chosen from which to make a prediction. The state of the system at that time step corresponds to a ‘focal point’ in the multi‐dimensional space. The set (called the library) of candidate nearest neighbours to the focal point is constructed, to determine the nearest neighbours that are then used to make the prediction. Our mathematical explanation explicitly documents which points in the multi‐dimensional space should not be considered as focal points. We suggest a new option for excluding points from the library that may be useful for short‐term time series that are often found in ecology. We focus on the core simplex and S‐map algorithms of EDM. Our new R package, pbsEDM, enhances understanding (by outputting intermediate calculations), reproduces our results and can be applied to new data. Our work improves the clarity of the inner workings of EDM, a prerequisite for EDM to reach its full potential in ecology and have wide uptake in the provision of advice to managers of natural resources.

## Graphical abstract

We provide a detailed introduction to empirical dynamic modelling (EDM), an increasingly popular method for understanding the dynamics of ecosystems. Our work provides greater clarity of the inner workings of EDM, a prerequisite for EDM to reach its full potential in ecology and have wide uptake in the provision of advice to managers of natural resources.

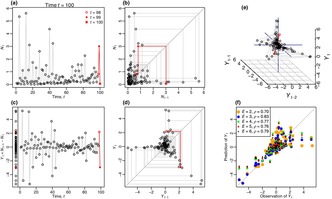

## 1 INTRODUCTION

Population forecasts are widely used to provide advice to natural resource managers and to understand how ecosystems might respond to a changing environment. They are usually made by prescribing a mathematical model to describe the system, fitting the model to data to estimate parameters and iterating the model forward in time, possibly under a range of future scenarios. For single populations, such models include Ricker models (e.g. Dorner et al., 2009), matrix population models (e.g. Caswell et al., 1999) and age‐structured models that require complex likelihood functions and computational Bayesian techniques to fit to data (e.g. Cleary et al., 2019). Multi‐trophic models of the marine ecosystem can consist of several coupled differential equations (e.g. Fasham et al., 1990).

Ecologists do have basic equations that might be considered as laws, such as a population will exponentially grow (or decline) under a constant environment (Turchin, 2001). However, even simple single‐species models consisting of a single difference equation can display qualitatively different behaviour with just slight changes in parameters (May, 1976). Multi‐trophic models of the marine ecosystem also exhibit such behaviour, yet it is hard to prescribe specific values to many parameters, such as growth and mortality (Edwards, 2001; Yool, 1998). Even slight structural changes in model formulation can also drastically change predictions (Wood & Thomas, 1999).

Empirical dynamic modelling (EDM) aims to avoid such issues by not requiring a mathematical model of the system being considered (Chang et al., 2017; Deyle et al., 2013; Munch et al., 2020; Sugihara & May, 1990; Ye et al., 2015). Therefore, there is no need to estimate parameters. Rather, EDM uses only the available data to calculate forecasts. It does this by translating time series of data into a path through multi‐dimensional space and making forecasts based on nearest spatial neighbours.

Recent examples demonstrate EDM's continuing application to fundamental problems in ecology. Ushio (2022) proposed a new hypothesis of how patterns of community diversity emerge, based on EDM analysis of environmental DNA data from experimental rice plots in Japan. Deyle et al. (2022) improved the quantitative understanding of food‐web and chemical changes in Lake Geneva by using a novel hybrid combination of a parametric physical model and an EDM analysis of the biogeochemical variables. Rogers et al. (2022) demonstrated that chaos is not rare in natural ecosystems by analysing a global database of single‐species population time series. Grziwotz et al. (2018) revealed complex environmental drivers of nine mosquito sub‐populations in French Polynesia. Karakoç et al. (2020) investigated community assembly and stability through an EDM analysis of populations of laboratory microbial communities. They found changes in species interactions that were driven by the presence of a predator, behaviour that is difficult to model with traditional Lotka‐Volterra‐type ecological models.

Such dependence of dynamics on another variable (predators in this case) is particularly amenable to analysis using EDM because EDM does not require explicitly prescribing equations, from the many choices available, to model such processes (Ye et al., 2015). As such, there may be a role for EDM in Ecosystem Based Fisheries Management, a current focus of several government agencies that takes into account the ecosystem when managing fisheries (Howell et al., 2021). Applications to fish populations have already been widespread, including cod (Sguotti et al., 2020), salmon (Ye et al., 2015) and tuna (Harford et al., 2017), plus forage fish such as menhaden, sardine and anchovy (Deyle et al., 2018; Sugihara et al., 2012). In simulation studies, EDM was found to provide low errors in forecasted fish recruitment (Van Beveren et al., 2021).

Here we expand upon the current literature that describes EDM, e.g. Chang et al. (2017), Deyle et al. (2013), Munch et al. (2020), Sugihara and May (1990), Ye et al. (2015), the third of which includes a useful glossary; see Munch et al. (2022) for an overview of recent advances in EDM, including methods for dealing with missing data. We build up a comprehensive description of the core simplex algorithm of EDM that explicitly gives the steps involved; such steps have probably not previously been described in such detail (G. Sugihara, Scripps Institution of Oceanography, pers. comm.). We use three progressively more detailed approaches, starting with a short verbal explanation. Next we give a graphical explanation (without all the precise details) for a simple example time series. Then we extend existing notation and derive the mathematics for the univariate situation, building on the explanation by Deyle et al. (2013). Technical mathematical concepts are kept to a minimum. We extend our derivation to the multivariate situation and the S‐map algorithm in Appendix S1.

Our motivation to obtain a deeper understanding of EDM originated in our desire to investigate the potential of using EDM to provide advice to fisheries managers, particularly in the context of considering ecosystem effects. We wanted to fully understand the methods so that we could write our own R package, pbsEDM (Rogers & Edwards, 2023), tailored to our specific applications. Despite the widespread use of EDM, we did not find a full description that explained all the steps unambiguously in sufficient detail for us to write our own independent code.

This lack of explanatory detail led to us developing the descriptions presented here to help users, particularly new ones, understand the inner workings of EDM. These descriptions include two aspects of EDM that we had not seen previously reported (though some practitioners may well be aware of them). We explicitly define the allowable focal points from which predictions can be made (aspect 1), and calculate the library (or set) of candidate nearest neighbours to use for predictions (aspect 2). This allows for a clearer understanding of how the size of the library depends on both the number of lags being considered in an analysis and on the time step from which a prediction is being made.

Note that EDM has been called ‘an equation‐free approach’ (Ye et al., 2015) due to it not specifying equations that represent a mathematical model to represent the system. The equations we introduce here do not represent a model, but explicitly and unambiguously explain the inner workings of EDM. The mathematical details themselves are not overly technical, mainly dealing with careful definitions of vectors and matrices, which first requires thoughtful consideration of notation, as is often the case (Edwards & Auger‐Méthé, 2019).

Practitioners of EDM should be aware that it has a long and strong theoretical background (Kantz & Schreiber, 2004; Packard et al., 1980; Schaffer & Kot, 1986; Stark et al., 2003; Takens, 1981), but do not need to understand the full details.

In our examples, the variables represent population numbers and associated environmental variables, but the methods are applicable to time series of any quantities in ecology or other fields. This does require that the system is not completely stochastic, and has some underlying deterministic rules (that may still be subject to some randomness). Our intention is for our pbsEDM R package to complement the popular R package rEDM (Park et al., 2023, with a tutorial at https://github.com/SugiharaLab/rEDM/blob/master/vignettes/rEDM‐tutorial.pdf) and Python package pyEDM (Park & Smith, 2023), to aid understanding and reproducibility. All intermediate calculations are available as output in pbsEDM and all code is in R, while rEDM contains C++ code (which is faster than R code but less readable than R to many ecologists); however, rEDM and pyEDM also include advanced algorithms that are not in pbsEDM. All code for reproducing our calculations and figures (each as a single function), and for applying methods to users' own data, is publicly available within pbsEDM (and File S1).

## 2 EXPLAINING EDM VERBALLY

The idea behind EDM is that we start with a simple time series of a variable, such as the annual values of the size of a population. We then construct additional time series of first‐differenced values (differences between consecutive values) and lags of those values (which compare the first‐differenced values with those in the past, such as 1 year previously or 2 years previously). These first‐differenced and lagged values then make up the components of vectors that can be plotted as points in a multi‐dimensional space known as a *state space*. Joining these points together in temporal order traces a path through the state space. To make a prediction from a point in the state space, the simplex algorithm finds the nearest neighbours (in terms of spatial distance in the state space) and sees where those neighbours went in their subsequent time step. A weighted average of these destinations yields the prediction. The key concept of EDM is the transforming of the time series of single values at each point in time, into points that lie in the multi‐dimensional state space. The points in the state space can reveal a geometric structure that is not apparent when viewing the data as a simple time series.

The reason that EDM can work is because the lagged values contain intrinsic information concerning the system. Ye et al. (2015) described the general concept as “local neighborhoods (and their trajectories) in the reconstruction [our state space] map to local neighborhoods (and *their* trajectories) of the original system”.

To expand on our brief verbal description, we now work through an analysis of an example time series using graphical explanations and then derive an explicit mathematical description of the simplex algorithm.

## 3 EXPLAINING EDM GRAPHICALLY

### 3.1 Plotting values from a simple time series in various ways

**FIGURE 1:**
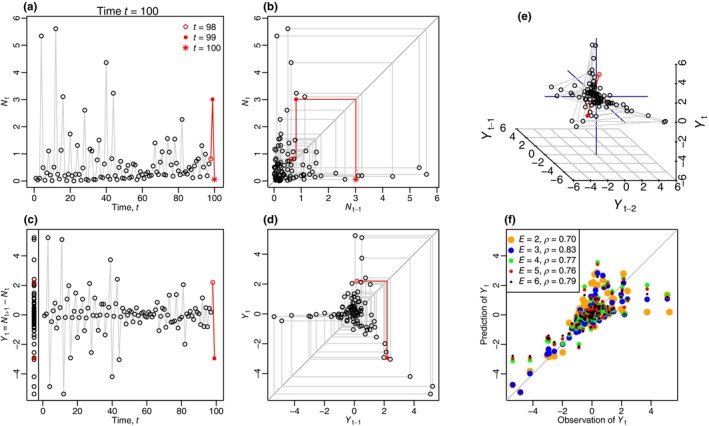
Different ways of plotting a simple simulated time series. Panels are arranged so that the y axes are the same for (a) and (b), and then for (c), (d) and (f). (a) Population values $N_{t}$ (units of 100,000 individuals) are shown through time. The final time step (t=100) is shown in the title, and the final three values of $N_{t}$ are shown in red using the symbols indicated (these carry through to the other panels except (f)). (b) Same values in a phase plane, with $N_{t}$ against $N_{t-1}$. The grey lines demonstrate the points progressing clockwise around the phase plane (see text). (c) Time series of resulting first‐differenced values $Y_{t}=N_{t+1}-N_{t}$, with all values also overlaid in a single column to the left of t=0. (d) Phase plane of $Y_{t}$ against $Y_{t-1}$ reveals a geometric structure that is not apparent in the preceding panels. (e) Extends (d) to three dimensions, showing $Y_{t}$, $Y_{t-1}$ and $Y_{t-2}$. (f) The predicted results and corresponding Pearson correlation coefficients, ρ, of the observed values of $Y_{t}$ for different values of embedding dimension E. Figure A.1: Appendix S1 shows a controllable frame‐by‐frame animation of this figure for t=1 to t=100, and Movie S1 gives a narrated version.

We start with a simple example time series of simulated data generated from a stochastic salmon population model that gives populations at times t=1,2,3,…,100. The time series is used for illustrative purposes, not to make any claims about applying EDM to any particular scenario. Figure 1 shows how the data can be plotted in five different ways, to introduce the concepts underpinning EDM.

Figure 1a shows the simple time series of the size of the population at time t, $N_{t}$, with generally low and occasionally high values. In Figure 1b the same data are shown with $N_{t}$ plotted against the value at the previous time step, namely $N_{t-1}$. This is a phase plane because there are two axes that represent variables and there is no time axis. Figure 1c introduces the first‐differenced values, defined as the difference between the population at the next time step and the current time step, that is, $Y_{t}=N_{t+1}-N_{t}$. By definition, $Y_{t}$ can take negative and positive values (while $N_{t}\geq 0$ since it represents the size of a population).

Figure 1d shows the phase plane of each $Y_{t}$ against its lagged value $Y_{t-1}$, revealing some geometric structure in the data. This structure is inherently coming from the population $N_{t}$ being mostly at low levels, but with occasional high values followed by immediate drops back down to low levels. There is clearly a cluster of points around the origin, representing low values of $Y_{t}$ (and $Y_{t-1}$) for most of the time series, due to small changes between consecutive values of $N_{t}$. There are three ‘arms’ along which the remaining points lie, plus empty areas of the phase plane that never appear to be visited. The central top arm contains points representing values of $Y_{t-1}$ close to zero that are then followed by a large value of $Y_{t}$ (a large increase in the population). When t=98 the system is at the location $\left(Y_{t-1},Y_{t}\right)=\left(Y_{97},Y_{98}\right)$, as indicated by the red open circle in Figure 1d. At the next time step, t=99, the system moves to $\left(Y_{98},Y_{99}\right)$, given by the red closed circle. A graphical way to view this is shown by the red lines in Figure 1d. First, trace horizontally from $\left(Y_{97},Y_{98}\right)$ to the 1:1 line $Y_{t-1}=Y_{t}$, so that the previous $Y_{t}$ value on the *y*‐axis ($Y_{98}$) becomes the new $Y_{t-1}$ value on the *x*‐axis (when t increases by 1, the old $Y_{t}$ becomes the new $Y_{t-1}$). Then trace up or down to reach the new $Y_{t}$ value. This approach, inspired by the dynamical systems concept of ‘cobwebbing’ (Murray, 1989), leads to the tracing out of the path of the grey lines in Figure 1d. This path is always clockwise. For the full time series, this results in the bottom‐right arm, for which a large value of $Y_{t-1}$ is always immediately followed by a large negative $Y_{t}$ (a large decline). Continuing clockwise leads to the left arm, for which the large declines are followed by very minor changes close to zero, and so in the next time step the trajectory heads back into the central cluster.

The cobwebbing idea graphically shows that, for example, when the population experiences a large increase (large $Y_{t}$), the next few time steps are expected to follow a certain path clockwise around the phase plane (namely, a large decrease in $Y_{t}$ shown in the bottom‐right arm, followed by a small value of $Y_{t}$ close to zero in the left arm). The idea of EDM is to harness such geometric structure in the spatial phase plane to make predictions in the time dimension.

Figure 1d also shows empty regions that the system does not visit, namely the top‐right area (a large increase of $N_{t}$ is never followed by another large increase: $Y_{t-1}$ and $Y_{t}$ are never both large), the bottom‐left area (a decline or slight increase is never followed by a large decline: $Y_{t-1}$ and $Y_{t}$ are never both very negative), and the top‐left area (a large decline is never followed by a large increase: a very negative $Y_{t-1}$ is never followed by a large $Y_{t}$). This last description translates to $N_{t}$ never going high, then low, then immediately high again.

The structure in Figure 1d represents the attractor on which the system evolves through time. This gives a useful way of thinking about EDM – the fundamental description of the dynamics of the system can be thought of as being given by the observed attractor (based solely on the data), rather than by a prescribed set of equations (Munch et al., 2020).

Figure 1e extends the two‐dimensional phase plane idea to three dimensions, showing $Y_{t}$ against $Y_{t-1}$ and $Y_{t-2}$. While Figure 1d included a lag of one time step, Figure 1e includes lags of one ($Y_{t-1}$) and two ($Y_{t-2}$) time steps. Again, this reveals an underlying geometric structure of the system (seen more clearly in the animated Figure A.1: Appendix S1 which shows the structure being built up through time). The three dimensions correspond, in EDM language, to an *embedding dimension* of E=3, because the points are embedded in three‐dimensional space. This space is known as the *state space* (the multi‐dimensional equivalent of the two‐dimensional phase plane). The phase plane in Figure 1d corresponds to E=2. Higher embedding dimensions (4, 5, 6, etc.) are also used, but obviously not easily plotted. The points at the left of Figure 1c show the distribution of values of $Y_{t}$ in one dimension, which is essentially an embedding dimension of E=1. This is not commonly used in EDM but is shown here to illustrate how we can have the points on a line for E=1, on a phase plane (Figure 1d) for E=2 and a three‐dimensional plot (Figure 1e) for E=3.

In hindsight, some of the aforementioned conclusions from Figure 1d can be teased out from Figures 1a,c, but the phase plane in (d) makes them much more apparent. However, EDM utilises structure in higher dimensions (i.e. using more lags) that cannot be easily visualised and cannot be inferred from the simple time series. Certainly, the structure in the three‐dimensional Figure 1e cannot be easily ascertained from the simple time series.

Figures 1a–e have simply plotted the data in different ways, there have been no statistical analyses or calculations beyond first‐differencing and lagging. Such plotting has revealed some structure behind the time series that is not immediately apparent in the simple time series plots. Figure 1f is discussed after we explain how EDM uses the geometric structure to make predictions.

### 3.2 Graphically demonstrating the simplex algorithm

For our example time series, we first choose a focal time $t^{*}$, which means that we want to use EDM to predict where the system goes in the subsequent time step (Deyle et al., 2013). We choose, as an example, $t^{*}=39$, such that we want to estimate $Y_{40}$ (where the system goes in the next time step) given knowledge of the rest of the time series. We denote the estimated value as ${\hat{Y}}_{40}$, and more generally, for a given $t^{*}$ we want to estimate ${\hat{Y}}_{t^{*}+1}$.

**FIGURE 2:**
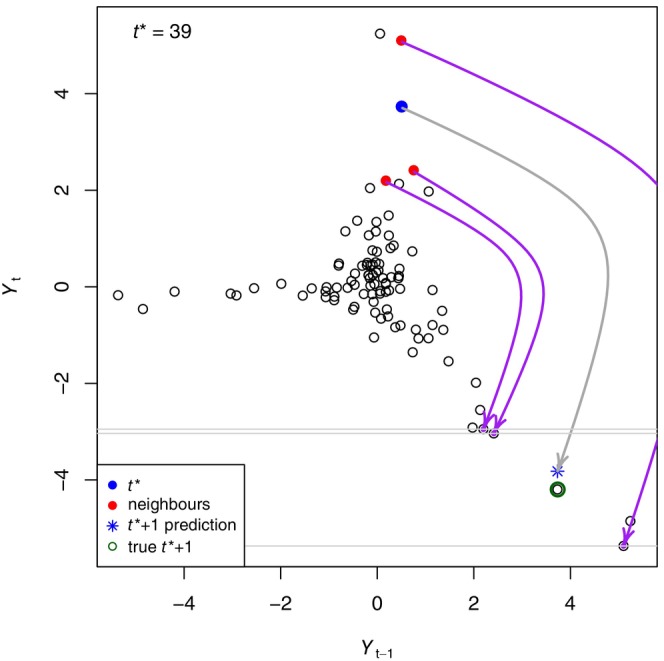
For the example time series, embedding dimension E=2 yields the phase plane of $Y_{t}$ against $Y_{t-1}$ as in Figure 1d. Given focal time $t^{*}=39$ (blue circle indicating $Y_{38}$ and $Y_{39}$) we want to predict $Y_{t^{*}+1}=Y_{40}$. The three nearest neighbours to the blue circle in the phase plane are shown by the red circles. These are at times 11, 43 and 98, though the times cannot be inferred from the phase plane. The purple arrows show where these points move to in the phase plane one time step later, namely to the points corresponding to times 12, 44 and 99. A weighted average of $Y_{12},Y_{44}$ and $Y_{99}$ (grey horizontal lines) then gives the estimate of $Y_{40}$ (blue star). In this case it is close to the known true value of $Y_{40}$ (green circle). An annotated animation of this figure is shown in Figure A.2: Appendix S1, and the figures for all valid $t^{*}$ values are shown in Figure A.3: Appendix S1.

We can then compare the predicted value ${\hat{Y}}_{40}$ to its known value to see how well the simplex algorithm performs for $t^{*}=39$. The state of the system at $t^{*}=39$ is highlighted in Figure 2, showing the values of $Y_{t-1}=Y_{38}$ and $Y_{t}=Y_{39}$ in the lagged phase plane. For the phase plane the nearest three neighbours are located (red circles). These are the nearest neighbours spatially, but this does not mean that they are close to each other in time; the actual times of these points are t=11,43, and 98. The crux of EDM is to see where these points move to in the phase plane in their next time step, to make a prediction of where the focal point will go. The idea being that close points in the phase plane will move to close points for their subsequent time step, and this structure in the system allows us to estimate ${\hat{Y}}_{t^{*}+1}={\hat{Y}}_{40}$.

The purple arrows in Figure 2 show where the three nearest neighbours move to in their subsequent time steps t=12,44, and 99 (recall from the cobwebbing idea that the $Y_{t}$ values become the new $Y_{t-1}$ values, and so it is only the new $Y_{t}$ values that give new information). A weighted average of these new $Y_{t}$ values then gives our prediction of ${\hat{Y}}_{t^{*}+1}={\hat{Y}}_{40}$ (the blue star), which here is close to the value of $Y_{40}$ already known from our time series (green circle). The weighting is based on the relative closeness of the three nearest neighbours to the focal point (explained in detail later).

We can make similar predictions for all alternative values of the focal time $t^{*}$ (in addition to $t^{*}=39$), and evaluate how the predicted values ${\hat{Y}}_{t^{*}+1}$ compare to the known values $Y_{t}$. We then calculate the Pearson correlation coefficient (ρ) of these, which is the usual, but not the only, way to characterise the performance of EDM predictions (Ye et al., 2015), with ρ=1.0 representing a perfect positive correlation between observations and predictions. For the phase plane from Figure 2, which has embedding dimension E=2, we have ρ=0.70.

**FIGURE 3:**
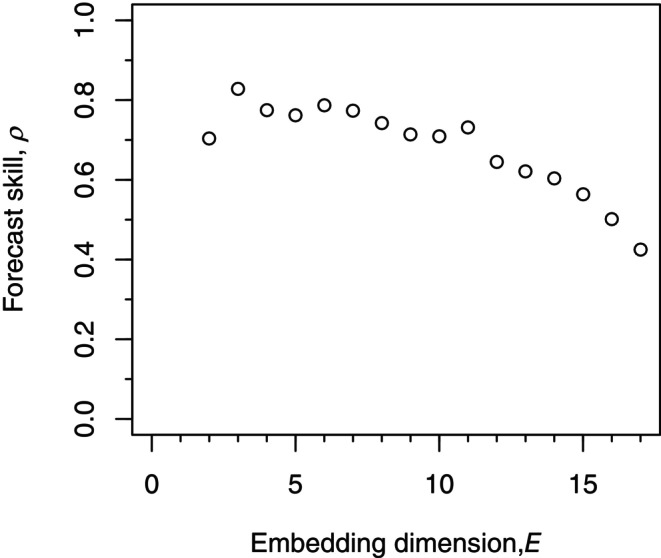
Dependence of the Pearson correlation coefficient, ρ, on embedding dimension, E, for the example time series from Figure 1. The best fitting model is given by the highest ρ, and corresponds to E=3 which is what would be used to forecast ${\hat{N}}_{T+1}$. We have shown high enough values of E to show a clear decline in ρ, but in general the maximum E considered should be about $\sqrt{T}$ (Munch et al., 2020), which is 10 here.

This idea is then repeated for prescribed embedding dimensions of E=3,4,5,…, and the predicted and observed values up to E=6 are shown in Figure 1f, together with the corresponding ρ. The best‐performing (highest ρ) embedding dimension is E=3 (Figure 3), and this is the dimension that would, therefore, be used in EDM to forecast the population into the future, beyond the timespan of the data.

## 4 EXPLAINING EDM MATHEMATICALLY

We now develop the above ideas using a more formal mathematical approach. We mostly follow and adapt the description given by Deyle et al. (2013), extending their work to give a full mathematical description that leads to a deeper understanding of EDM and its limitations. The simplex algorithm is described for a univariate time series, such as an annual survey estimate of a population, and extended to the multivariate case and the S‐map technique (Sugihara, 1994) in Appendix S1. Notation is extended from that defined clearly by Deyle et al. (2013), and summarised in Table 1 for reference.

**TABLE 1:** The main notation used here.

Notation	Definition
*Indices*	
t	Index for time; t=1,2,3,…,T
T	Number of time steps
$t^{*}$	Focal time at which we know the state of the system and want to predict the state at $t^{*}+1$
*Variables*	
$N_{t}$	Value, such as population size, at t=1,…,T
$Y_{t}$	First‐difference value $Y_{t}=N_{t+1}-N_{t}$
$\hat{\cdot }$	Estimate of ⋅
*EDM calculations*	
${\tilde{x}}_{t}$	Vector of length E *defining* the axes of the lagged state space, for example, ${\tilde{x}}_{t}=\left(Y_{t},Y_{t-1},Y_{t-2}\right)$
$x_{t}$	*Realised values* of the components of ${\tilde{x}}_{t}$, for example, $x_{t}=\left(3,-5,1\right)$; each element of $x_{t}$ is the value along each axis of the E‐dimensional space, where the axes are defined by components of ${\tilde{x}}_{t}$
$x_{t^{*}}$	*Realised values* of the components of ${\tilde{x}}_{t}$ at the focal time $t=t^{*}$
E	Embedding dimension, the number of dimensions of the state space in which the system is being embedded to look for the nearest E+1 neighbours to the focal point $x_{t^{*}}$; E is the length of ${\tilde{x}}_{t}$
X	Matrix with rows representing time and columns representing each of the E components of ${\tilde{x}}_{t}$; row t represents the system state at time t with the *j*th element representing the *j*th component of $x_{t}$
$ℒ\left(E,t^{*}\right)$	Library for a given E and $t^{*}$, consisting of the set of $x_{t}$ that are candidates to be considered as nearest neighbours of $x_{t^{*}}$
$C_{E,t^{*}}$	The number of vectors $x_{t}$ in the library $ℒ\left(E,t^{*}\right)$
$C_{E}$	The usual value of $C_{E,t^{*}}$ for a given E, defined as $C_{E}=T-2\left(E+1\right)$; $C_{E,t^{*}}\geq C_{E}$
$\psi_{i}$	After calculating the distance between $x_{t^{*}}$ and each $x_{t}$ in the library, $\psi_{1}$ gives the time index of the $x_{t}$ that is the nearest neighbour to $x_{t^{*}}$, $\psi_{2}$ corresponds to the second nearest neighbour, etc.

### 4.1 Algorithm for simplex projection

We consider a univariate time series of population size $N_{t}$ at each time t=1,2,3,…,T. As earlier, we first‐difference the data to give scalars

(1) $$Y_{t}=N_{t+1}-N_{t}$$

for t=1,…,T−1, such that the first value, $Y_{1}$, is defined, with $Y_{T}$ undefined. First‐differencing is often done to help remove any simple linear mean trend (Chang et al., 2017). The aim of the analysis is to estimate ${\hat{N}}_{T+1}$, that is, the population the year after the final year of data, by estimating ${\hat{Y}}_{T}$ and then rearranging (1) to give ${\hat{N}}_{T+1}={\hat{Y}}_{T}+N_{T}$.

The simplex algorithm was detailed as steps (i) to (vii) by Deyle et al. (2013). These are summarised and extended in Table 2 to give an overall idea of the approach and then expanded upon here.

**TABLE 2:** The steps of the simplex algorithm (extended from Deyle et al., 2013).

Step	Brief description
(i)	Translate the time series values into vectors in the multi‐dimensional state space defined by a given embedding dimension E
(ii)	Pick a focal time from which to predict
(iii)	Define the set of library vectors of candidate nearest neighbours to the focal point
(iv)	Calculate the distances between appropriate points in the state space
(v)	Identify the nearest neighbours to the focal point
(vi)	Make a prediction using a weighted average of the known next positions of the nearest neighbours
(vii)	Repeat steps (ii)–(vi) for all appropriate focal times
(viii)	Calculate the correlation coefficient between predictions and the known observations
(ix)	Repeat steps (i)–(viii) for different values of E, using the optimal one (E with maximum correlation coefficient) to forecast the future value of the population

(i) For a given embedding dimension E, we define the vector ${\tilde{x}}_{t}$ in lagged space as containing $Y_{t}$ and consecutive lags down to $Y_{t-E+1}$:

(2) $${\tilde{x}}_{t}=\left[\begin{array}{c} Y_{t}\\ Y_{t-1}\\ \ldots \\ Y_{t-E+2}\\ Y_{t-E+1} \end{array}\right].$$

So ${\tilde{x}}_{t}$ has length E with each element defining an axis that we will be using to construct the E‐dimensional state space. Actual realised values (numbers) for a particular t are recorded in vectors $x_{t}$, with each element referring to its corresponding axis definition in ${\tilde{x}}_{t}$. For example, with our simulated time series from Figure 1, E=4 yields

(3) $${\tilde{x}}_{t}=\left[\begin{array}{c} Y_{t}\\ Y_{t-1}\\ Y_{t-2}\\ Y_{t-3} \end{array}\right]\;\;\text{yielding}\;\;x_{4}=\left[\begin{array}{c} Y_{4}\\ Y_{3}\\ Y_{2}\\ Y_{1} \end{array}\right]=\left[\begin{array}{c} -4.854\\ 5.241\\ 0.059\\ -0.057 \end{array}\right],x_{5}=\left[\begin{array}{c} Y_{5}\\ Y_{4}\\ Y_{3}\\ Y_{2} \end{array}\right]=\left[\begin{array}{c} -0.461\\ -4.854\\ 5.241\\ 0.059 \end{array}\right],\ldots ,$$

with ${\tilde{x}}_{t}$ defining the axes of the state space.

The components of ${\tilde{x}}_{t}$ from (3) give the column headings of matrix X:
(4)

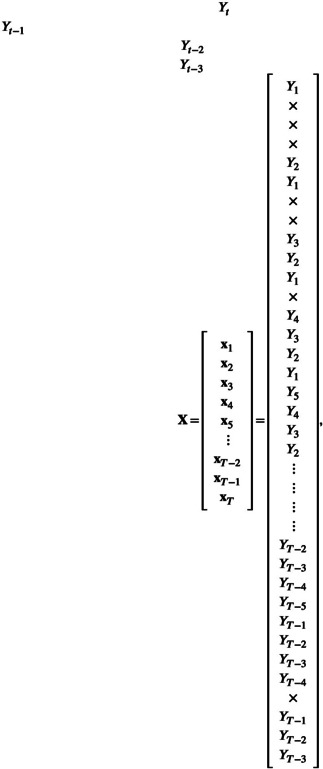

for which row t consists of the explicit values of $x_{t}$ (implicitly understood to be written here as a row vector) with undefined values indicated by ×. Vectors $x_{1},x_{2}$ and $x_{3}$ are undefined because the $Y_{t}$ for t≤0 are undefined. Some brief descriptions of the simplex algorithm do not mention that some points should be excluded from the library (e.g. Hsieh et al., 2005; Ye et al., 2015), while Deyle et al. (2013) did note that the first few time values will not have a vector in the state space; here we make that more explicit. Also, $x_{T}$ does not exist because $Y_{T}$ is undefined in (1); however, we include it in X because we will want to forecast $Y_{T}$ and it is helpful for X to have T rows.

Matrix (4) is for E=4. Extending this for a general value of E we have
(5)

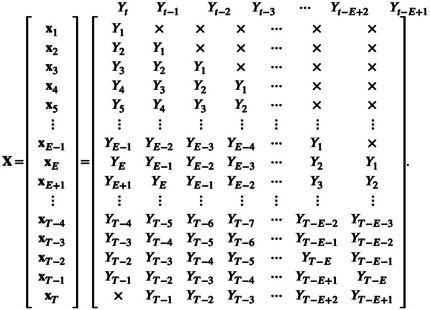




For a given time series of length T, the larger the value of E, the larger the size of the upper‐right triangle of undefined values, because a larger embedding dimension requires more lagged values. The first row that is fully known (requiring that $Y_{t-E+1}$ exists) is when t=E.

(ii) Pick a focal time $t^{*}$ for which we know $Y_{t^{*}}$ and want to predict the value of $Y_{t^{*}+1}$, with the prediction denoted ${\hat{Y}}_{t^{*}+1}$. In the E‐dimensional state space, we do this by requiring knowledge of the full $x_{t^{*}}$ and then estimating ${\hat{x}}_{t^{*}+1}$ to give us our estimate of ${\hat{Y}}_{t^{*}+1}$ from (2). Not all values of t are available to use for $t^{*}$ (aspect 1; briefly explained in Table 3), which will be made explicit shortly. Note that we call ${\hat{Y}}_{t^{*}+1}$ for general $t^{*}$ a ‘prediction’, reserving the term ‘forecast’ for estimating future ${\hat{Y}}_{T}$ and ${\hat{N}}_{T+1}$ beyond the existing data.

**TABLE 3:** Two aspects of EDM that we document here to aid new users of EDM.

Aspect	Brief description
1	The allowable focal point times $t^{*}$ (from which to make predictions) depend explicitly upon the embedding dimension E. They require lagged values that do not extend before the start of the time series or beyond the end of it
2	We explicitly calculate the library of candidate nearest neighbours of the focal point, and derive a new relationship showing how the size of the library depends on both $t^{*}$ and E

(iii) Given $t^{*}$, define the library $\left\{x_{t}\right\}$ of candidate nearest neighbours of $x_{t^{*}}$. To determine the library $\left\{x_{t}\right\}$ we start with an expanded version of X from (5) for general $t^{*}$, and systematically cross out $Y_{t^{*}+1}$ and various $x_{t}$ that must be excluded from the library due to four conditions, resulting in

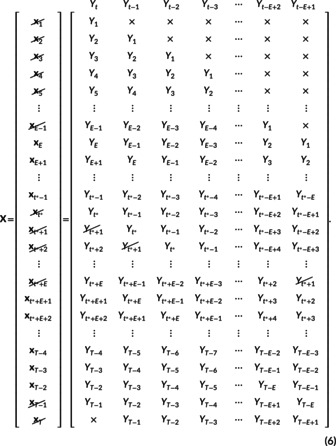

The four conditions for excluding components from the library of candidate nearest neighbours are:

- $x_{t^{*}}$ cannot be a nearest neighbour to itself—excludes $x_{t^{*}}$;
- Several $x_{t}$ are not fully defined (contain ×)—excludes $x_{1},x_{2},\ldots ,x_{E-1}$ and $x_{T}$;
- Exclude any t for which we do not know $x_{t+1}$ (since we need to know where the nearest neighbours go in the subsequent time step in (vi))—excludes $x_{T-1}$;
- It may not be appropriate to use any $x_{t}$ that includes $Y_{t^{*}+1}$, since we are trying to predict $Y_{t^{*}+1}$—excludes $x_{t^{*}+1},x_{t^{*}+2},\ldots ,x_{t^{*}+E}$. This is further investigated later.

**FIGURE 4:**
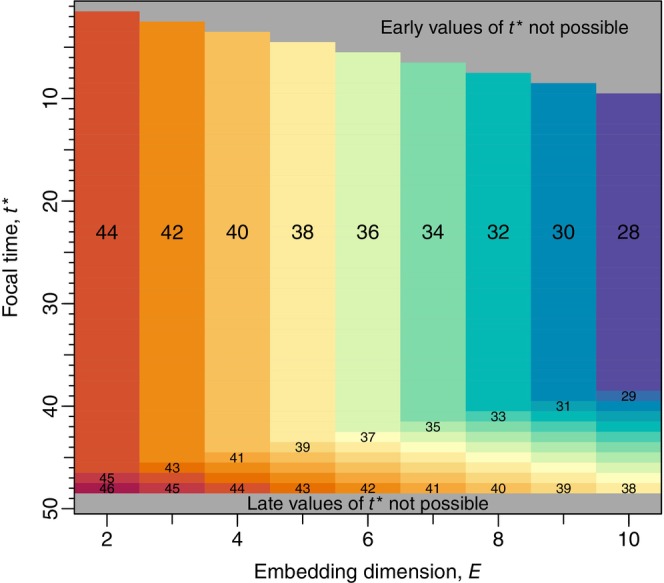
The number of components of the library, $C_{E,t^{*}}$, depends on the interplay between embedding dimension E and focal time $t^{*}$, as derived in (11) and shown here for any univariate time series of length T=50. The $t^{*}$ axis is inverted to compare with (6). The top grey area indicates $t^{*}$ values that are not possible because the required lagged values (based on E) extend before the start of the time series. The bottom grey area indicates $t^{*}$ values that are not possible because for t=50 the first‐difference value is not known, and for t=49 the predicted state at t=50 is not known (and we first want to compare predictions to known values.) For each value of E, the majority of $C_{E,t^{*}}$ values are $C_{E}=T-2\left(E+1\right)$, as shown by the large‐font numbers (indicating $C_{2}=44$, $C_{3}=42$ etc.; see later text) that define the colours. Colours increment by one going down the figure, as illustrated by the small‐font numbers. For E=8, for example, $t^{*}$ can only take values between 8 and 48, and the library size is usually 32, but incrementally increases from 33 to 40 as $t^{*}$ increases from 41 to 48.

The resulting library is given by the remaining set of $x_{t}$ vectors that are not crossed out in (6), namely:

(7) $$ℒ\left(E,t^{*}\right)=\left\{x_{E},x_{E+1},\ldots ,x_{t^{*}-2},x_{t^{*}-1},x_{t^{*}+E+1},x_{t^{*}+E+2},\ldots ,x_{T-2}\right\},$$

for those $x_{t}$ that are defined, where the notation $ℒ\left(E,t^{*}\right)$ emphasises that the library depends upon both E and $t^{*}$; this is aspect 2. For a time series of length T=50, Figure 4 shows how the size of the library, $C_{E,t^{*}}$, varies with E and $t^{*}$.

Returning to the idea from (ii) of determining the valid values of $t^{*}$, we now flesh out aspect 1, determining the allowable values of $t^{*}$ and how these depend on E. Matrix X in (6) shows that $x_{t}$ is not defined for t=1,2,…,E−1, such that $t^{*}$ cannot take these values; this is the top‐right grey region of Figure 4. Also, $x_{T}$ is not defined and so $t^{*}$ cannot equal T. The value $t^{*}=T-1$ is also excluded because in step (viii) we want to compare the prediction with the known state, which cannot be done for $t^{*}=T-1$ since $x_{t^{*}+1}=x_{T}$ is not defined. Though note that $t^{*}=T-1$ can later be used to forecast ${\hat{x}}_{T}$ and hence ${\hat{Y}}_{T}$ and ${\hat{N}}_{T+1}$, which is the aim of the analysis. Excluding T and T−1 corresponds to the bottom grey region in Figure 4, leaving allowable values for the focal time $t^{*}$ of E,E+1,…,T−3,T−2, which are the non‐grey combinations in Figure 4.

Usually, the library has $C_{E}=T-2\left(E+1\right)$ components, as indicated by the bulk of values for each value of E in Figure 4 (e.g. $C_{2}=44$, since T=50). Intuitively, the library has fewer components as E gets larger because the larger E uses more temporal lags which creates a higher dimensional state space, resulting in X having more columns and subsequently more $x_{t}$ crossed out in (6) and more grey area at the top (small $t^{*}$ values) of Figure 4.

However, the library size also depends on $t^{*}$, and we denote it by $C_{E,t^{*}}$. We now derive $C_{E,t^{*}}$, as used to create Figure 4. For a given E, $C_{E,t^{*}}>C_{E}$ for certain values of $t^{*}$, because for larger $t^{*}$ the crossed out $x_{t^{*}+1},x_{t^{*}+2},\ldots ,x_{t^{*}+E}$ components in the middle rows of (6) overlap with the crossed out $x_{T-1}$ and $x_{T}$ components, or do not exist since they have times >T. This overlapping first happens when $t^{*}+E=T-1$, such that $t^{*}=T-E-1$ and the library is

(8) $$ℒ\left(E,t^{*}\right)=ℒ\left(E,T-E-1\right)=\left\{x_{E},x_{E+1},\ldots ,x_{T-E-3},x_{T-E-2}\right\},$$

which has size $C_{E,t^{*}}=C_{E,T-E-1}=T-E-2-\left(E-1\right)=C_{E}+1$. In Figure 4, this corresponds to $C_{2,47}=45$ (for E=2) and $C_{3,46}=43$ (for E=3).

For the next value, $t^{*}=T-E$, we have

(9) $$ℒ\left(E,T-E\right)=\left\{x_{E},x_{E+1},\ldots ,x_{T-E-2},x_{T-E-1}\right\},$$

which has size $C_{E,t^{*}}=C_{E,T-E}=T-E-1-\left(E-1\right)=C_{E}+2$, corresponding to $C_{2,48}=46$ and $C_{3,47}=44$ in Figure 4.

This pattern incrementally increases until we get to $t^{*}=T-2$, near the end of the time series, for which $x_{t^{*}+1}$ and $x_{T-1}$ are the same. So in (6), $x_{T-1}$ gets excluded both because we do not know $x_{T}$ (exclusion condition c) and because it contains $Y_{t^{*}+1}$ (condition d). This overlap means that the library is

(10) $$ℒ\left(E,T-2\right)=\left\{x_{E},x_{E+1},\ldots ,x_{T-4},x_{T-3}\right\},$$

which has size $C_{E,T-2}=T-3-\left(E-1\right)=C_{E}+E$. In Figure 4, for E=2 this is the aforementioned $C_{2,48}=46$, and for E=3, this is, $C_{3,48}=45$.

In summary, the library is given by (7) and is bigger for relatively large $t^{*}$, with size explicitly given by
(11)

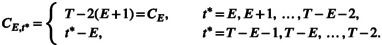




It was previously noted that (for a given E) the library will consist of all possible vectors formed from the time series, except for the target vector (Deyle et al., 2013). However, here we have shown that other vectors also need to be excluded and that the library size also depends explicitly on $t^{*}$ (that we have not seen stated previously). In our example, this means that for E=8 the library size can vary from 32 to 40 depending on the focal time (Figure 4).

(iv) The next step is to calculate the Euclidean distance in the state space between the focal point $x_{t^{*}}$ and each point in the library. The Euclidean distance between two vectors $a=\left(a_{1},a_{2},\ldots ,a_{E}\right)$ and $b=\left(b_{1},b_{2},\ldots ,b_{E}\right)$ is defined as

(12) $$∥a-b∥={\left[{\left(a_{1}-b_{1}\right)}^{2}+{\left(a_{2}-b_{2}\right)}^{2}+\ldots +{\left(a_{E}-b_{E}\right)}^{2}\right]}^{1/2}.$$

(v) Rank every vector in the library with respect to its Euclidean distance from $x_{t^{*}}$, and define $\psi_{i}$ to be the time index of the vector with rank i. So the nearest neighbour has rank 1 and will have time index $\psi_{1}$, the second nearest will have rank 2 and time index $\psi_{2}$, etc. The closest state‐space vectors $x_{\psi_{1}}$ and $x_{\psi_{2}}$ indicate points in the library for which the system was in the most similar state to the focal time $t^{*}$, for this particular state‐space reconstruction (value of E). Of interest are the E+1 nearest neighbours, which form a simplex in the E‐dimensional state space (hence the ‘simplex algorithm’). A simplex in an E‐dimensional space consists of E+1 points (a triangle for E=2, a tetrahedron or triangular pyramid for E=3, etc.). In Figure 2, for which $t^{*}=39$, the nearest E+1=3 neighbours (red points) are at times $\psi_{1}=43,\psi_{2}=11$ and $\psi_{3}=98$. Thus, based on $Y_{t}$ and its lagged value $Y_{t-1}$, the system appears closest to its state at time 39 at times 43, 11 and 98 (times that are not necessarily close to 39, but the system is similar in the state space); this is the core concept of EDM, and is certainly not discernible from viewing the data as a simple time series.

(vi) In one time step, each vector $x_{\psi_{i}}$ moves to its corresponding location $x_{\psi_{i}+1}$. We use the nearest E+1 neighbours (so i=1,2,…,E+1) and take a weighted average of the first components of the resulting $x_{\psi_{i}+1}$. By definition from (6), $x_{t^{*}+1}=\left[Y_{t^{*}+1},Y_{t^{*}},\ldots ,Y_{t^{*}-E+2}\right]$; it is only the first component of this vector that we are estimating (the other components are already known). Hence the weighted average only concerns the first component of the nearest‐neighbour vectors, namely the $Y_{\psi_{i}+1}$. We make a prediction ${\hat{Y}}_{t^{*}+1}$ for $Y_{t^{*}+1}$ using equation S1 from Deyle et al. (2013):

(13) $${\hat{Y}}_{t^{*}+1}=\frac{\sum_{i=1}^{E+1}w_{i}Y_{\psi_{i}+1}}{\sum_{j=1}^{E+1}w_{j}},$$

where the weights $w_{i}$ are

(14) $$w_{i}=\exp\left(-\frac{∥x_{t^{*}}-x_{\psi_{i}}∥}{∥x_{t^{*}}-x_{\psi_{1}}∥}\right).$$

The weights downweight the contribution of each $Y_{\psi_{i}+1}$ based on the closeness of $x_{\psi_{i}}$ to $x_{t^{*}}$ relative to the closeness of $x_{\psi_{1}}$ (the closest vector) to $x_{t^{*}}$; note that Deyle et al. (2013) had the above summations to E, but they should be to E+1 for the E+1 nearest neighbours, as in Sguotti et al. (2020). By definition, the weight of the closest vector is always $w_{1}=\exp\left(-1\right)=0.368$.

(vii) For short time series (like our example) cross‐validation is used to test how well the method performs on the known data. This involves repeating steps (ii)‐(vi) with all valid values of $t^{*}$ for which we can compare the observed $Y_{t^{*}+1}$ with the predicted ${\hat{Y}}_{t^{*}+1}$. Longer time series can be split to use the first half to predict the second half (Deyle et al., 2013).

(viii) Determine the correlation coefficient, ρ, between the observed $Y_{t^{*}+1}$ and predicted ${\hat{Y}}_{t^{*}+1}$, defined as

(15) $$\rho =\frac{\sum_{t^{*}}\left(Y_{t^{*}+1}-\bar{Y}\right)\left({\hat{Y}}_{t^{*}+1}-\bar{\hat{Y}}\right)}{\sqrt{\sum_{t^{*}}{\left(Y_{t^{*}+1}-\bar{Y}\right)}^{2}\cdot \sum_{t^{*}}{\left({\hat{Y}}_{t^{*}+1}-\bar{\hat{Y}}\right)}^{2}}}$$

where $\bar{Y}=\text{mean}\;\left(Y_{t^{*}+1}\right)$ and $\bar{\hat{Y}}=\text{mean}\;\left({\hat{Y}}_{t^{*}+1}\right)$, and these means, and the summations in (15) are over the valid values of $t^{*}$ (as in Figure 4).

(ix) Repeat steps (i) to (viii) for a sequence of embedding dimensions E. The E that gives the highest ρ is considered to perform best, namely E=3 (giving ρ=0.83) for our example time series (Figure 3). That E is used to forecast the future value of the population, ${\hat{N}}_{T+1}={\hat{N}}_{101}$, by setting $t^{*}=99$ to estimate ${\hat{Y}}_{100}$ and rearranging (1) to give ${\hat{N}}_{101}={\hat{Y}}_{100}+N_{100}$. Note that, regarding aspect 1, $t^{*}=T-1=99$ is allowed here for forecasting ${\hat{N}}_{101}$; its exclusion in (6) is only for determining ρ. If ρ increases with E such that there is no optimal E, this suggests a high‐dimensional essentially random process for all practical purposes, such that the system is difficult to model (Hsieh et al., 2005).

For our simulated data and E=3, our pbsEDM implementation of steps (i)–(ix) gives ${\hat{Y}}_{100}=-0.077$ yielding ${\hat{N}}_{101}=-0.077+0.060=-0.017$. Thus, the forecast is of a negative population, which is obviously unrealistic. Predictions of the first‐differenced ${\hat{Y}}_{t}$ are weighted averages of observed values of $Y_{t}$, so they must lie within the range of the observed values (e.g. Figure 2). More extreme values are not possible. But there is nothing to stop the resulting ${\hat{N}}_{t}$ predictions being more extreme than for the observed values of $N_{t}$, which includes allowing negative values. Negative values are predicted for six ${\hat{N}}_{t}$ in our example time series (see File S1). We suggest the simple remedy of replacing the negative predictions with the smallest observed value from the original $N_{t}$ time series. A second option is to replace $N_{t}$ with $\log\;N_{t}$ (which can be negative), although results will differ because relative distances of nearest neighbours will change, altering the weights in (14); Rogers et al. (2022) implemented both $N_{t}$ and $\log\;N_{t}$. A third option is to not first‐difference the original data (discussed below).

Relatedly, we find ρ=0.83, but calculating the correlation based on $N_{t}$ and ${\hat{N}}_{t}$ instead, by replacing Y with N in (15), gives 0.54; for E=2 we get 0.70 and 0.28. Thus, we caution that high correlation based on $Y_{t}$ does not necessarily imply high correlation based on $N_{t}$, which is what we are interested in (see below and File S1).

Condition (d) above is that it may be appropriate to exclude $x_{t^{*}+1},x_{t^{*}+2},\ldots ,x_{t^{*}+E}$ from the library of candidate nearest neighbours of the focal point $x_{t^{*}}$. This is based on the principle that when testing the predictive accuracy of a method it is problematic to use information about the value being predicted. The method should not have any knowledge of the known value of the quantity.

For our simulated data and E=2, we find that predictions ${\hat{Y}}_{t^{*}+1}$ are the same when using pbsEDM or rEDM, except for $t^{*}=75$ (0.838 for pbsEDM versus 1.368 for rEDM) and $t^{*}=94$ (0.412 versus 0.177). For $t^{*}=75$, we find that rEDM uses $x_{t^{*}+1}=x_{76}=\left(Y_{76},Y_{75}\right)$ as one of the three nearest neighbours to $x_{75}$, and hence uses it in the prediction ${\hat{Y}}_{76}$, despite it including $Y_{76}$ (which is what we are trying to predict). We find this by changing the value of $Y_{76}$ to a large value such that $x_{76}$ is no longer a close neighbour of $x_{75}$, and the rEDM code then gives the exact same answer as for pbsEDM (also agreeing with some earlier code that we wrote independently of pbsEDM); see File S1.

Similarly, for $t^{*}=94$ we find that rEDM uses $x_{t^{*}+2}=x_{96}=\left(Y_{96},Y_{95}\right)$ as a nearest neighbour of $x_{94}$, but this neighbour includes the value of $Y_{95}$ that we are trying to predict (and we suggest it should be excluded). The default in rEDM is to not exclude any temporally adjacent neighbours, although the exclusionRadius argument allows the user to exclude nearest temporal neighbours within exclusionRadius time steps of $t^{*}$ (i.e. this would exclude the exclusionRadius number of $x_{t}$ both above and below $x_{t^{*}}$ in (6), which can help deal with autocorrelation). For short time series as we have in our fisheries applications, we would like to retain as many potential neighbours as possible, and so in pbsEDM our default is as described above in (6) and (7), and we also provide options to match the settings from rEDM. Differences between such options will become more important for higher embedding dimensions than 2, since the excluded points $x_{t^{*}+1},x_{t^{*}+2},\ldots ,x_{t^{*}+E}$, become more numerous as E increases.

Note that forecasting ${\hat{N}}_{T+1}$ involves setting $t^{*}=T-1$, for which the excluded points just referred to would be the undefined $x_{T},x_{T+1},\ldots ,x_{T-1+E}$. So although the different options will not directly affect the nearest neighbours of $x_{T-1}$ and the ${\hat{N}}_{T+1}$ calculation, they do affect the calculation of ρ and hence the choice of E used for forecasting, which can indeed influence ${\hat{N}}_{T+1}$.

For our simulated data the largest $Y_{t}$ is $Y_{3}$ for t=3 (Figure 1c). Predicting ${\hat{Y}}_{3}$ requires $t^{*}=2$ which is valid for E=2 but no higher E (Figure 4). Yet $Y_{3}$ is the poorest estimated value of all (being the right‐most point of Figure 1f). So the most poorly estimated point is included in the ρ calculations only for E=2, which seems an unfair constraint when comparing ρ for different E (Figure 3) to find the optimal E to use for forecasting. Future investigations could examine whether restricting calculations to the same set of $t^{*}$, based on Figure 4, should be done when determining the optimal E.

Whether to apply the first‐differencing or not will be time‐series dependent. Chang et al. (2017) state that linear trends in the original data should be removed, either by simple regression or taking the first‐difference, to make the time series stationary. First‐differencing was not strictly necessary for our example time series (there was no clear linear trend in the $N_{t}$), yet the first‐differenced lagged values in Figure 1d do demonstrate geometric structure that is not seen in the non‐first‐differenced values in Figure 1b. Our explanations are the same without first‐differencing, with $Y_{t}$ simply taking the value $N_{t}$ instead of $N_{t+1}-N_{t}$. Real applications can test sensitivity to first‐differencing.

In Appendix S1 we extend the above mathematical description to the multivariate situation of analysing multiple variables, such as populations of several species or a population and an index of local temperature. The library of candidate nearest neighbours to the focal point can again be calculated. The size of the library does not depend on the chosen embedding dimension, just on the maximum lag, m, used for any of the variables. The size is once more represented by Figure 4, but with the E‐axis replaced by m+1. So the library size does not change if further variables are added unless they are lagged more than the existing variables such that m increases. We describe the S‐map algorithm in Appendix S1 and apply it to our simulated data set.

## 5 DISCUSSION

We have derived a thorough description of the core methods of EDM, yielding previously undocumented aspects that improve understanding. Having gained a deeper understanding of EDM, our work suggests potential enhancements. For example, the closest E+1 neighbours are typically selected for the simplex algorithm (to form a simplex in the E‐dimensional space), but simulations could investigate how altering the numbers of neighbours (an easily changed parameter in rEDM) might improve accuracy. This could lead to developing a bootstrapping approach to produce confidence intervals for simplex predictions. Simulation testing could determine the observed coverage of such intervals (and also for bootstrap intervals from the S‐map algorithm, as used by Karakoç et al., 2020).

Readers searching the literature should be aware of other terms that describe EDM‐type approaches, including nonlinear forecasting, state‐space reconstruction, Takens' theorem, time‐delay embedding and Jacobian Lyapunov exponents. To delve into the more technical background behind EDM, we recommend the books by Ott et al. (1994), particularly Chapter 5 on ‘The Theory of Embedding’ and the included reprints of Sauer (1993) and Sugihara and May (1990), and Huffaker et al. (2017), particularly Chapter 3 on ‘Phase Space Reconstruction’.

The use of EDM can allow for time‐varying productivity (or other ecosystem changes) to be implicitly accounted for in applications such as fisheries management. For example, Ye et al. (2015) found that including time series of sea surface temperature when forecasting salmon populations using EDM performed better than not including temperature, and that EDM outperformed parametric models. Fruitful research could further compare EDM with parametric time‐varying models (for which it is necessary but hard to prescribe an explicit mathematical relationship between productivity and time). How this would directly inform decision‐making requires further investigation, since, in general, accounting for nonstationarity in the ecosystem requires careful consideration of how to determine the benchmarks or reference points that are used to determine the status of stocks (Holt & Michielsens, 2020). So although we have described EDM as an alternative to parametric mechanistic modelling, both approaches can be used together in various complementary ways (Munch et al., 2020), and this may indeed be how EDM fulfils its potential in practical management applications.

## AUTHOR CONTRIBUTIONS

**Andrew M. Edwards:** Conceptualization (equal); data curation (equal); formal analysis (lead); funding acquisition (equal); investigation (equal); methodology (equal); project administration (equal); software (equal); supervision (equal); validation (lead); visualization (lead); writing – original draft (lead); writing – review and editing (lead). **Luke Rogers:** Data curation (equal); formal analysis (supporting); investigation (equal); methodology (equal); software (equal); writing – review and editing (supporting). **Carrie Holt:** Conceptualization (equal); data curation (equal); formal analysis (supporting); funding acquisition (equal); investigation (equal); methodology (equal); project administration (equal); supervision (equal); writing – original draft (supporting); writing – review and editing (supporting).

## Supporting information


Appendix S1

Movie S1

File S1

Data S1

## Data Availability

No new data were collected. All results are reproducible from the File S1.
