# Supplementary material for: Explaining empirical dynamic modelling using verbal, graphical and mathematical approaches
Source: Ecol Evol. 2024 May 15;14(5):e10903. doi: 10.1002/ece3.10903 (PMC11094587; doi:10.1002/ece3.10903)
Supplement: Supplementary file 1 — Appendix S1 [file ECE3-14-e10903-s003.pdf]

Appendix S1: further figures and calculations

Appendix for

'Explaining empirical dynamic modelling using verbal, graphical and mathematical approaches'

by Andrew M. Edwards, Luke A. Rogers, and Carrie A. Holt. *Ecology and Evolution*. DOI: 10.1002/ece3.10903

This Appendix first contains the following animations, each of which is controllable by the user (pause, skip forward and back, etc.):

- Figure A.1: Animated version of Figure 1, with one frame for each value of time t.
- Figure A.2: Animated annotated version of Figure 2 for the focal time $t^* = 39$, graphically explaining the main steps of the simplex algorithm.
- Figure A.3: Animation showing Figure 2 but with each frame corresponding to a value of the focal time $t^* = 2, 3, 4, ..., 99$.

Note that animation controls may not work in all pdf readers (Adobe Acrobat Reader is fine). Figure A.1 is also presented in Supplementary Movie S1 as a short narrated version.

Then follows the mathematical description of the simplex algorithm for multivariate time series, expanding on the description in the main text for univariate time series. We then describe the S-map algorithm.

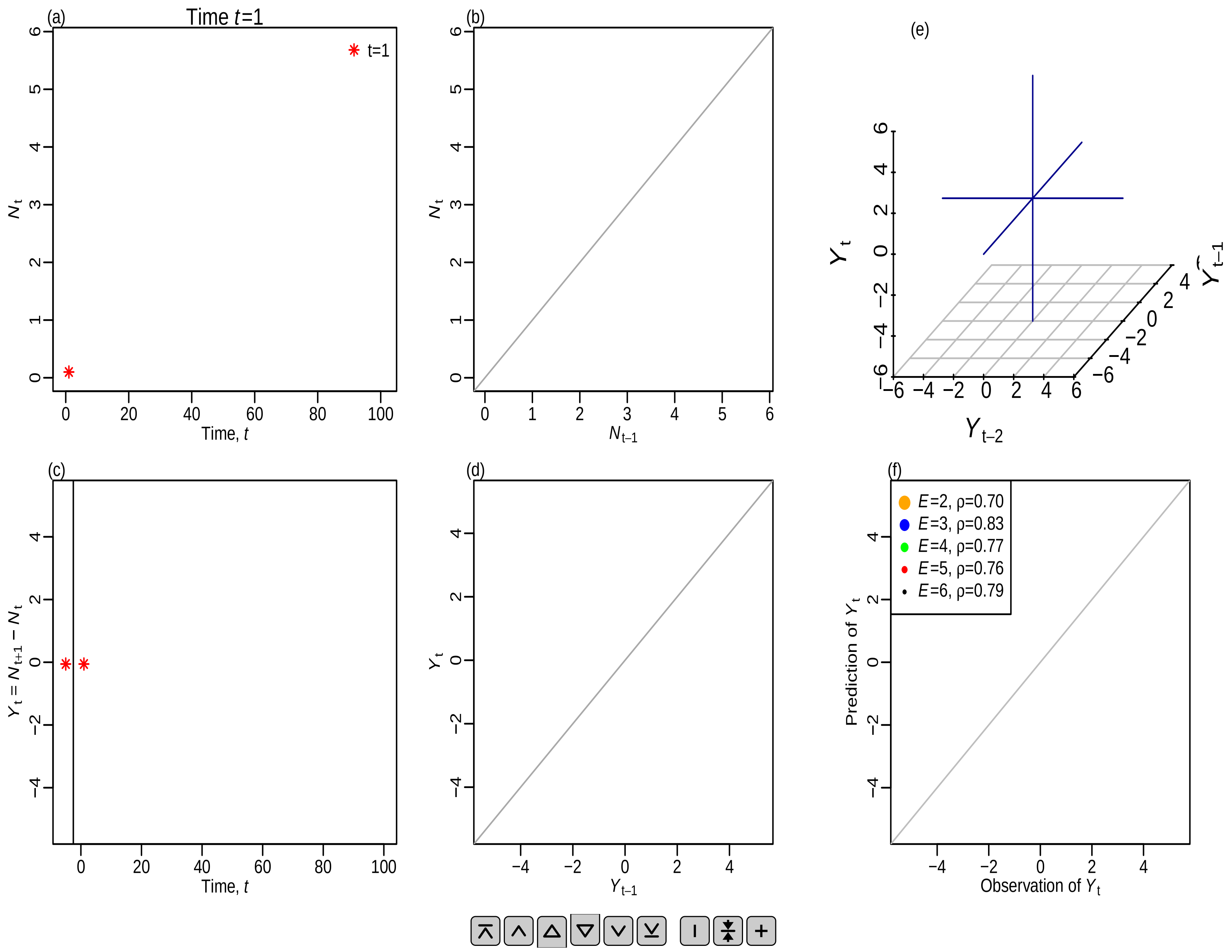

Figure A.1: Animated version of Figure 1, controllable using the buttons (should start automatically; pause then click < or > to manually go through at own pace). A narrated version is given in Movie S1.

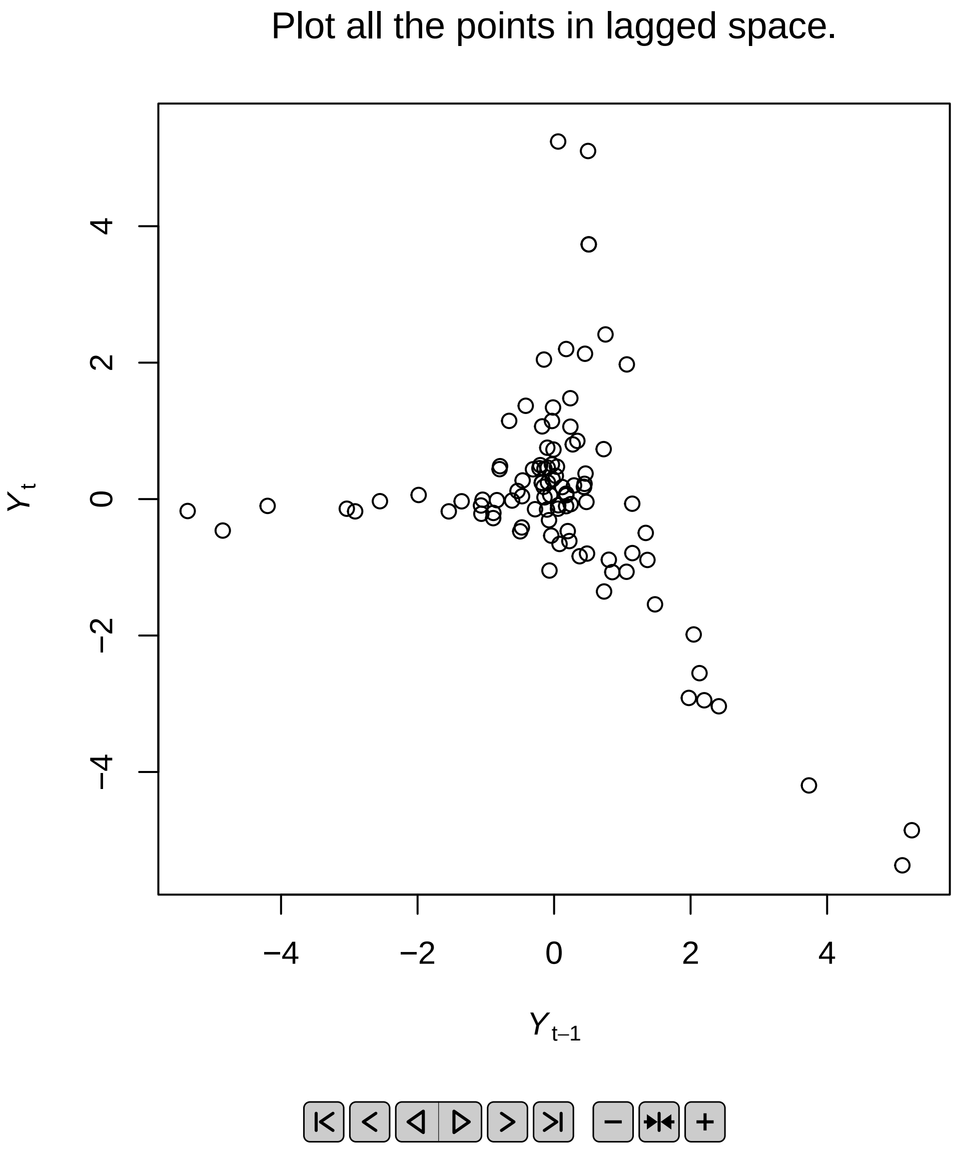

Figure A.2: Animated annotated version of the individual steps of Figure 2.

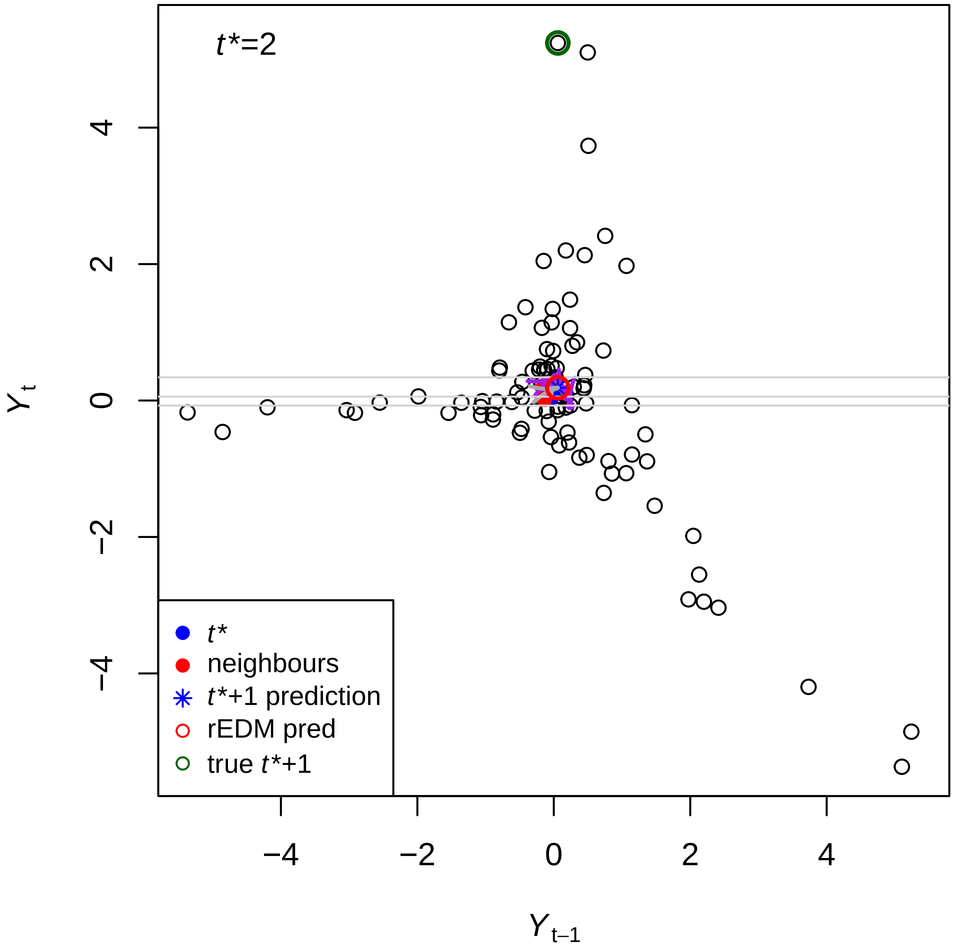

Figure A.3: Animated version of Figure 2 for each valid value of t^*. Good predictions of Y_{t^*+1} involve the blue asterisk lying within (or close to) the green open circle; red open circle shows the `rEDM` prediction. Interesting values that help understanding of EDM are: $t^* = 2, 10, 14, 38$ (poor prediction of the subsequent large Y_{t^*+1}, though $t^* = 42$ predicts its large Y_{t^*+1} well); $t^* = 3, 11, 15, 43$ (from the top 'arm' the predictions are generally good); $t^* = 4, 12, 16, 44$ (from the bottom-right 'arm' the predictions are generally good); $t^* = 5, 13, 17, 45$ (from the left 'arm' the predictions are generally good though $t^* = 13$, the leftmost point, is not great); $t^* = 47, 70$ (two of the nearest neighbours go in completely opposite directions); $t^* = 34, 46, 64, 88$ (one or two nearest neighbours gives high Y_t, erroneously giving a large prediction); $t^* = 75, 94$ (discrepancy with `rEDM` calculation, see main text); $t^* = 10, 80$ (all neighbours move somewhat close by but the true Y_{t^*+1} jumps more). This animation helps understand how certain dynamics (large negative Y_t) are much better predicted than others (large positive Y_t); depending on the application this asymmetry may be important to consider. The understanding of the predictions of the 'arms' gives intuition as to how adding more lags (higher E) can help predictions.

Table A.1: The main extra notation for the multivariate simplex and the S-map algorithms.

**Notation**	**Definition**
	**Indices**
k	Index for variables; $k = 1, 2, 3, ..., K$
K	Number of variables
	**Variables**
$N_{t,k}$	Time t value of variable k
$Y_{t,k}$	First-differenced and standardised value of variable k at time t
$\mathbf{N}$	Matrix of data with elements $N_{t,k}$
$\mathbf{Z}$	Intermediate matrix of first-differenced values, $Z_{t,k} = N_{t+1,k} - N_{t,k}$
$\mathbf{Y}$	Matrix of first-differenced and standardised values with elements $Y_{t,k}$ defined in (A.3)
	**Simplex calculations**
$\tilde{\mathbf{x}}_t$	Vector *defining* the axes of the lagged state space, for example $\tilde{\mathbf{x}}_t = (Y_{t,1},\ Y_{t-1,1},\ Y_{t,2})$
m	Maximum lag of all variables used in $\tilde{\mathbf{x}}_t$
C'_{m,t^*}	For a given m and t^*, the number of vectors $\mathbf{x}_t$ in the library of candidate nearest neighbours of $\mathbf{x}_{t^*}$
	**S-map calculations**
θ	Degree of local weighting used for the S-map algorithm.

Mathematical explanation for multivariate time series

Now consider K time series of variables to be analysed together (extra notation used here is summarised in Table A.1). Simple examples would be time series of prey and predator populations, or a population and a temperature index, or a single species in different spatial locations. The appeal of EDM is that explicit mathematical relationships between, say, temperature and a population do not need to be prescribed from the choice of many plausible relationships. We denote

$N_{t,k}$ as the element at time t of time series k, giving the matrix

$$\mathbf{N} = \begin{bmatrix} N_{1,1} & N_{1,2} & \cdots & N_{1,K} \\ N_{2,1} & N_{2,2} & & \\ \vdots & & \ddots & \\ N_{T,1} & & & N_{T,K} \end{bmatrix}, \tag{A.1}$$

such that each column represents the known time series of variable k. We designate the first column, for which $k = 1$, to be the main variable of interest (such as an animal population) that we wish to forecast based on the influence of the other variables (such as air or sea surface temperature); thus the aim is to forecast $\hat{N}_{T+1,1}$.

For the univariate time series we used the first-differences of values. For multivariate time series there is also a need to then standardise the values such that each variable is on a similar scale (Chang *et al.*, 2017). For brevity we include this in the calculation of $\mathbf{Y}$, with the first-differencing calculated as

$$Z_{t,k} = N_{t+1,k} - N_{t,k} \tag{A.2}$$

for $t = 1, 2, ..., T-1$, and the standardising (centering and scaling) by subtracting each first-differenced variable by its mean, μ_k, and dividing by its standard deviation, σ_k:

$$Y_{t,k} = \frac{Z_{t,k} - \mu_k}{\sigma_k}, \tag{A.3}$$

where

$$\mu_k = \frac{1}{T-1} \sum_{i=1}^{T-1} Z_{i,k}, \tag{A.4}$$

$$\sigma_k^2 = \frac{1}{T-2} \sum_{i=1}^{T-1} \left(Z_{i,k} - \mu_k\right)^2. \tag{A.5}$$

This defines

$$\mathbf{Y} = \begin{bmatrix} Y_{1,1} & Y_{1,2} & \cdots & Y_{1,K} \\ Y_{2,1} & Y_{2,2} & & \\ \vdots & & \ddots & \\ Y_{T-1,1} & & & Y_{T-1,K} \end{bmatrix}. \tag{A.6}$$

Note that it is important to first-difference and then standardise (not vice versa).

We now generalise the steps (i)-(ix) that were described for the univariate case in Table 3 and associated text.

(i) For a given number of variables K and embedding dimension $E > K$, there is now a choice of axes that can be used to construct the state space. For example, with $K = 2$ and $E = 3$ there are two possible definitions of the components of $\tilde{\mathbf{x}}_t$:

$$\tilde{\mathbf{x}}_t = \begin{bmatrix} Y_{t,1} \\ Y_{t-1,1} \\ Y_{t,2} \end{bmatrix} \quad \text{or} \quad \tilde{\mathbf{x}}_t = \begin{bmatrix} Y_{t,1} \\ Y_{t,2} \\ Y_{t-1,2} \end{bmatrix}. \tag{A.7}$$

Both definitions necessarily contain the unlagged variables $Y_{t,1}$ and $Y_{t,2}$, because we need to know where the system is at time t, while the first definition contains the first variable with a lag of 1 (i.e. $Y_{t-1,1}$) and the second contains the second variable with a lag of 1 (i.e. $Y_{t-1,2}$).

Similarly, with $E = 4$ the choices for $\tilde{\mathbf{x}}_t$ are

$$\begin{bmatrix} Y_{t,1} \\ Y_{t-1,1} \\ Y_{t-2,1} \\ Y_{t,2} \end{bmatrix}, \begin{bmatrix} Y_{t,1} \\ Y_{t-1,1} \\ Y_{t,2} \\ Y_{t-1,2} \end{bmatrix}, \begin{bmatrix} Y_{t,1} \\ Y_{t,2} \\ Y_{t-1,2} \\ Y_{t-2,2} \end{bmatrix}. \tag{A.8}$$

With $K = 4$ variables and $E = 6$, there are 10 options in all for $\tilde{\mathbf{x}}_t$:

$$\begin{bmatrix} Y_{t,1} \\ Y_{t-1,1} \\ Y_{t-2,1} \\ Y_{t,2} \\ Y_{t,3} \\ Y_{t,4} \end{bmatrix}, \begin{bmatrix} Y_{t,1} \\ Y_{t-1,1} \\ Y_{t,2} \\ Y_{t-1,2} \\ Y_{t,3} \\ Y_{t,4} \end{bmatrix}, \begin{bmatrix} Y_{t,1} \\ Y_{t-1,1} \\ Y_{t,2} \\ Y_{t,3} \\ Y_{t-1,3} \\ Y_{t,4} \end{bmatrix}, \begin{bmatrix} Y_{t,1} \\ Y_{t-1,1} \\ Y_{t,2} \\ Y_{t,3} \\ Y_{t,4} \\ Y_{t-1,4} \end{bmatrix}, \begin{bmatrix} Y_{t,1} \\ Y_{t,2} \\ Y_{t-1,2} \\ Y_{t-2,2} \\ Y_{t,3} \\ Y_{t,4} \end{bmatrix}, \begin{bmatrix} Y_{t,1} \\ Y_{t,2} \\ Y_{t-1,2} \\ Y_{t,3} \\ Y_{t-1,3} \\ Y_{t,4} \end{bmatrix}, \begin{bmatrix} Y_{t,1} \\ Y_{t,2} \\ Y_{t-1,2} \\ Y_{t,3} \\ Y_{t,4} \\ Y_{t-1,4} \end{bmatrix}, \begin{bmatrix} Y_{t,1} \\ Y_{t,2} \\ Y_{t,3} \\ Y_{t-1,3} \\ Y_{t-2,3} \\ Y_{t,4} \end{bmatrix}, \begin{bmatrix} Y_{t,1} \\ Y_{t,2} \\ Y_{t,3} \\ Y_{t-1,3} \\ Y_{t,4} \\ Y_{t-1,4} \end{bmatrix}, \begin{bmatrix} Y_{t,1} \\ Y_{t,2} \\ Y_{t,3} \\ Y_{t,4} \\ Y_{t-1,4} \\ Y_{t-2,4} \end{bmatrix} \quad \text{(A.9)}$$

So the various choices of $\tilde{\mathbf{x}}_t$ define the various state spaces. In practice the analyst will still need to make some up-front decisions so as to not have to explore too many choice of $\tilde{\mathbf{x}}_t$. All sensible combinations could be tested, and the one with maximum ρ used for forecasting.

(ii) Pick a focal time t^* for which we know $\mathbf{x}_{t^*}$ and a library of similar vectors. Estimating $\hat{\mathbf{x}}_{t^*+1}$ will give us our estimate of $Y_{t^*+1,k}$ for each k. We may not be interested in all components (we do not wish to predict next year's temperature using a salmon population model), but the values may still help in testing how well the state-space reconstruction can estimate known values.

(iii) Define the library of candidate nearest neighbours of $\mathbf{x}_{t^*}$. It is not feasible to write out the library, analogous to (7), for general K, E, t^* and choice of state-space axes (the components of $\tilde{\mathbf{x}}_t$). So here we derive $\mathbf{X}$ for the earlier $K = 4$ and $E = 6$ example, and use this to understand and derive the library size for the general case. Specifically we use the second choice of $\tilde{\mathbf{x}}_t$ from (A.9), namely all four variables at time t and lags of 1 for the first two variables, as shown in the

column headings here, to give:

$$
\mathbf{X} = \begin{bmatrix} \cancel{\mathbf{x}_1} \\ \mathbf{x}_2 \\ \mathbf{x}_3 \\ \mathbf{x}_4 \\ \mathbf{x}_5 \\ \mathbf{x}_{E=6} \\ \vdots \\ \mathbf{x}_{t^*-1} \\ \cancel{\mathbf{x}_{t^*}} \\ \cancel{\mathbf{x}_{t^*+1}} \\ \cancel{\mathbf{x}_{t^*+2}} \\ \mathbf{x}_{t^*+3} \\ \vdots \\ \mathbf{x}_{T-4} \\ \mathbf{x}_{T-3} \\ \mathbf{x}_{T-2} \\ \cancel{\mathbf{x}_{T-1}} \\ \cancel{\mathbf{x}_T} \end{bmatrix} = \begin{array}{c} \begin{array}{cccccc} \scriptstyle Y_{t,1} & \scriptstyle Y_{t-1,1} & \scriptstyle Y_{t,2} & \scriptstyle Y_{t-1,2} & \scriptstyle Y_{t,3} & \scriptstyle Y_{t,4} \end{array} \\ \left[\begin{array}{cccccc} Y_{1,1} & \times & Y_{1,2} & \times & Y_{1,3} & Y_{1,4} \\ Y_{2,1} & Y_{1,1} & Y_{2,2} & Y_{1,2} & Y_{2,3} & Y_{2,4} \\ Y_{3,1} & Y_{2,1} & Y_{3,2} & Y_{2,2} & Y_{3,3} & Y_{3,4} \\ Y_{4,1} & Y_{3,1} & Y_{4,2} & Y_{3,2} & Y_{4,3} & Y_{4,4} \\ Y_{5,1} & Y_{4,1} & Y_{5,2} & Y_{4,2} & Y_{5,3} & Y_{5,4} \\ Y_{6,1} & Y_{5,1} & Y_{6,2} & Y_{5,2} & Y_{6,3} & Y_{6,4} \\ \vdots & \vdots & \vdots & \vdots & \vdots & \vdots \quad \vdots \\ Y_{t^*-1,1} & Y_{t^*-2,1} & Y_{t^*-1,2} & Y_{t^*-2,2} & Y_{t^*-1,3} & Y_{t^*-1,4} \\ Y_{t^*} & Y_{t^*-1,1} & Y_{t^*,2} & Y_{t^*-1,2} & Y_{t^*,3} & Y_{t^*,4} \\ \cancel{Y_{t^*+1,1}} & Y_{t^*,1} & \cancel{Y_{t^*+1,2}} & Y_{t^*,2} & \cancel{Y_{t^*+1,3}} & \cancel{Y_{t^*+1,4}} \\ Y_{t^*+2,1} & \cancel{Y_{t^*+1,1}} & Y_{t^*+2,2} & \cancel{Y_{t^*+1,2}} & Y_{t^*+2,3} & Y_{t^*+2,4} \\ Y_{t^*+3,1} & Y_{t^*+2,1} & Y_{t^*+3,2} & Y_{t^*+2,2} & Y_{t^*+3,3} & Y_{t^*+3,4} \\ \vdots & \vdots & \vdots & \vdots & \vdots & \vdots \quad \vdots \\ Y_{T-4,1} & Y_{T-5,1} & Y_{T-4,2} & Y_{T-5,2} & Y_{T-4,3} & Y_{T-4,4} \\ Y_{T-3,1} & Y_{T-4,1} & Y_{T-3,2} & Y_{T-4,2} & Y_{T-3,3} & Y_{T-3,4} \\ Y_{T-2,1} & Y_{T-3,1} & Y_{T-2,2} & Y_{T-3,2} & Y_{T-2,3} & Y_{T-2,4} \\ Y_{T-1,1} & Y_{T-2,1} & Y_{T-1,2} & Y_{T-2,2} & Y_{T-1,3} & Y_{T-1,4} \\ \times & Y_{T-1,1} & \times & Y_{T-1,2} & \times & \times \end{array}\right] \end{array} . \quad \text{(A.10)}
$$

As for the univariate case, the four conditions for excluding components from the library of candidate nearest neighbours are:

(a) $\mathbf{x}_{t^*}$ cannot be a nearest neighbour to itself – excludes $\mathbf{x}_{t^*}$;

(b) some $\mathbf{x}_t$ are not fully defined (contain $\times$) – excludes $\mathbf{x}_1$ and $\mathbf{x}_T$ (note that this removes far less rows than for the univariate case);

(c) exclude any t for which we do not know $\mathbf{x}_{t+1}$ – excludes $\mathbf{x}_{T-1}$;

(d) it may not be appropriate to use any $\mathbf{x}_t$ that includes any $Y_{t^*+1,k}$, since we are trying to predict $Y_{t^*+1,1}$ and should not have knowledge of the covariates $Y_{t^*+1,2}, Y_{t^*+1,3}$, and $Y_{t^*+1,4}$ – excludes $\mathbf{x}_{t^*+1}$ and $\mathbf{x}_{t^*+2}$ (note that this also removes far less rows than for the univariate case).

The resulting library of candidate nearest neighbours is:

$$\{\mathbf{x}_2, \mathbf{x}_3, ..., \mathbf{x}_{t^*-1}, \mathbf{x}_{t^*+3}, \mathbf{x}_{t^*+4}, ..., \mathbf{x}_{T-2}\}, \tag{A.11}$$

which has size $T-6$ except for certain values of t^*.

Using a similar approach to the univariate case, we find that the the library size for the general multivariate case depends on the maximum lag, m, where $m = 1$ for the example in (A.10), as:

$$C'_{m,t^*} = \begin{cases} T - 2(m+2), & t^* = m+1, m+2, ..., T-m-3, \\ t^* - m - 1, & t^* = T-m-2, T-m-1, ..., T-2. \end{cases} \tag{A.12}$$

Note that this does not depend on the number of variables, K, or the embedding dimension, only on the maximum lag m. This means that adding more variables (more information) to the analysis will not reduce the library size if the maximum lag stays the same. Furthermore, (A.12) is equivalent to replacing E with $m+1$ in (11), which is consistent with the univariate case for which $E = m+1$. So a plot of C'_{m,t^*} is simply the same as Figure 4 with E replaced by $m+1$. The pattern of invalid t^* values and the library size depends only on m and not on the overall embedding dimension.

The actual embedding dimension E depends on the exact combination of lagged variables used (the choices in (A.9) all have $E = 6$ but either $m = 1$ or $m = 2$), and satisfies $E \geq K + m$. So although the embedding dimension will increase with extra variables (larger K), the library size will remain the same. And for a given embedding dimension E, the library is always larger for the multivariate case than for the univariate case (for which $E = m+1$), because the extra variables 'use up' some of the available embedding dimensions.

(iv)-(vii) These steps are the same as the univariate case, to rank the nearest neighbours to $\mathbf{x}_{t^*}$.

This is because once t^* and the library of valid vectors are defined, it does not matter whether the dimensions of the state space relate to multiple variables (multivariate case) or to just lagged values of one variable (univariate case), the calculations are the same. For (vi) the equivalent weighting is done for each $\hat{Y}_{t^*+1,k}$ (each k) that we want to predict (just $k = 1$ for a single population and environmental covariates, or multiple k if the variables represent multiple populations).

(viii) The calculation of ρ is the same as in (15) with Y_{t^*+1} replaced by $Y_{t^*+1,k}$ and $\hat{Y}_{t^*+1}$ replaced by $\hat{Y}_{t^*+1,k}$.

(ix) Repeating steps (i)-(viii) can be done for sensible definitions of $\tilde{\mathbf{x}}_t$ (not simply just different E, as for the univariate case). The highest correlation coefficient would then give the choice of $\tilde{\mathbf{x}}_t$ to use for forecasting $\hat{N}_{T+1,1}$.

S-map algorithm

Sugihara (1994) introduced the 'sequential locally weighted global linear map', or S-map for short. This has since been used by first obtaining the optimal embedding dimension E using the simplex algorithm, and then making predictions based on all the points in the state space, rather than just the $E+1$ nearest neighbours (e.g. Hsieh *et al.* 2005; Deyle *et al.* 2022). The same library of candidate nearest neighbours for the simplex algorithm becomes a library of all valid neighbours, again given by (7) with size C_{E,t^*} given by (11). Predictions are done by a weighting approach similar to (13) and (14) for the simplex algorithm, where θ represents the degree of local weighting, and the weights u_i are

$$u_i = \exp\left(-\frac{\theta \parallel \mathbf{x}_{t^*} - \mathbf{x}_{\psi_i} \parallel}{\bar{d}}\right), \tag{A.13}$$

where $\bar{d}$ is the mean distance of all the points in the library from the focal point:

$$\bar{d} = \frac{1}{C_{E,t^*}} \sum_{k=1}^{C_{E,t^*}} \parallel \mathbf{x}_{t^*} - \mathbf{x}_{\psi_k} \parallel . \tag{A.14}$$

A larger value of θ weights the closest points to $\mathbf{x}_{t^*}$ relatively more than the further points, such that predictions depend more strongly on the local region around $\mathbf{x}_{t^*}$. The extreme of $\theta = 0$ means that all weights are $u_i = 1$ and predictions are simply the global mean of the predicted values of the valid neighbours. This is a linear calculation, hence $\theta = 0$ represents a linear prediction, and increasing θ represents increasing nonlinearity (Hsieh *et al.*, 2005). The value of θ that yields the maximum of the correlation coefficient (ρ) is calculated and used for forecasting. To solve for the predictions requires use of singular value decomposition calculations (Hsieh *et al.*, 2005; Deyle *et al.*, 2022).

For our simulated data set with $E = 3$ (the optimal E from the simplex calculations) we get $\theta = 2.7$ for which $\rho = 0.87$, slightly higher than the $\rho = 0.83$ obtained for the simplex algorithm. For forecasting, we estimate $\hat{Y}_{100} = 0.480$, giving $\hat{N}_{101} = 0.480 + 0.060 = 0.541$ (rounded up). Thus, we avoid a negative forecast of the population (unlike for the simplex algorithm). However, for our full time series we do still obtain three negative predictions using S-map. Again, there is no guarantee that high correlation based on unlagged $\hat{Y}_t$ values also yields high correlation based on the $\hat{N}_t$ variables of interest.

The allowable values of t^* and consequent library of neighbours are the same as for the simplex algorithm, so the descriptions of aspects 1 and 2 occur for S-map. We find that the `rEDM` implementation of the S-map algorithm again differs slightly from ours, based on the default candidate neighbours to exclude. With $E = 3$, the optimal $\theta = 2.7$, and focal time $t^* = 93$, we estimate $\hat{Y}_{94} = 0.384$ using `pbsEDM` and 0.191 using `rEDM` (see the `pbsSmap` vignette in `pbsEDM`). However, by incorporating the values of Y_{92} to Y_{95} as dummy data for $t = 100$ to 104, we can 'fool' the `pbsEDM` S-map algorithm to allow the values $\mathbf{x}_{t^*+1}$, $\mathbf{x}_{t^*+2}$, and $\mathbf{x}_{t^*+3}$ to be included in the library of neighbours (because they are no longer close in time to $t^* = 93$). The `pbsEDM` S-map algorithm then estimates $\hat{Y}_{94} = 0.194$, which is very close to the `rEDM` estimate of 0.191 (they are not expected to be identical because the extra dummy points also affect $\bar{d}$).
