# Supplementary material for: Explaining empirical dynamic modelling using verbal, graphical and mathematical approaches
Source: Ecol Evol. 2024 May 15;14(5):e10903. doi: 10.1002/ece3.10903 (PMC11094587; doi:10.1002/ece3.10903)
Supplement: Supplementary file 3 — File S1 [file ECE3-14-e10903-s004.docx]

Supplementary File S1: pbsEDM-main.zip Archived version of our open-source pbsEDM R package. The package is freely available at https://github.com/pbs-assess/pbsEDM. It includes instructions for installation, plus links to the rendered vignettes used to demonstrate code and produce our results. All manuscript figures are reproducible with single functions listed in the README file.
