# Supplementary material for: Explaining empirical dynamic modelling using verbal, graphical and mathematical approaches
Source: Ecol Evol. 2024 May 15;14(5):e10903. doi: 10.1002/ece3.10903 (PMC11094587; doi:10.1002/ece3.10903)
Supplement: Supplementary file 4 — Data S1 [file ECE3-14-e10903-s001.zip › pbsEDM-main/vignettes/analyse_simple_time_series.html]

Analyse a Simple Time Series


# Analyse a Simple Time Series

```
library(pbsEDM)
```

This vignette shows how to use the simplex algorithm in `pbsEDM` to analyse a simple time series and forecast the next value, and shows resulting graphics that help understand the algorithm (as described in the manuscript). The tibble `NY_lags_example` is saved in the package and contains several columns, though we are only going to use the original time series of simulated population numbers, \(N\_t\), which has column name `N_t`. See `?NY_lags_example` for full details.

## Plot the data in different ways

We want to plot the data in different ways, for which it is easiest to first run the simplex algorithm (to create a `pbsEDM` object for which code automatically makes the figures).

Use only the `N_t` column from `NY_lags_example` and apply the simplex algorithm using lags of 0 and 1 (\(E = 2\)) with first differencing:

```
E_2_res <- pbsEDM(NY_lags_example,
                  lags = list(N_t = c(0:1)),
                  first_difference = TRUE)
```

This creates a `pbsEDM` object which is a list of results (see `?pbsEDM` for full details):

```
summary(E_2_res)
#>                    Length Class      Mode     
#> N                    101  -none-     numeric  
#> N_observed           101  -none-     numeric  
#> N_forecast           101  -none-     numeric  
#> Z                    100  -none-     numeric  
#> Z_observed           100  -none-     numeric  
#> Z_forecast           100  -none-     numeric  
#> Y                    100  -none-     numeric  
#> Y_observed           100  -none-     numeric  
#> Y_forecast           100  -none-     numeric  
#> X                    200  matrix     numeric  
#> X_observed           100  -none-     numeric  
#> X_forecast           100  -none-     numeric  
#> X_distance         10000  matrix     numeric  
#> neighbour_distance   300  -none-     numeric  
#> neighbour_index      300  -none-     numeric  
#> neighbour_value      300  -none-     numeric  
#> neighbour_weight     300  -none-     numeric  
#> projected_index      300  -none-     numeric  
#> projected_value      300  -none-     numeric  
#> projected_weight     300  -none-     numeric  
#> lags                   1  -none-     list     
#> p                      1  -none-     numeric  
#> first_difference       1  -none-     logical  
#> centre_and_scale       1  -none-     logical  
#> exclusion_radius       1  -none-     character
#> results                5  data.frame list
```

For now we’ll just use that to plot the data in various useful ways:

```
plot(E_2_res,
     portrait = FALSE)
```

The figure shows, before using the results of the simplex algorithm, the data in various ways: time series of \(N\_t\) and \(Y\_t\), plus phase plots of \(N\_t\) vs \(N\_{t-1}\), \(Y\_t\) vs \(Y\_{t-1}\) (as used for \(E=2\)) and \(Y\_t\) vs \(Y\_{t-1}\) vs \(Y\_{t-2}\) (as used for \(E=3\)). The last 3 values of \(N\_t\) are in red, with the final one as a star. In the \(Y\_t\) time series panel, the values of \(Y\_t\) are also plotted in a vertical line (to the left of \(t=0\)) which is the one-dimensional equivalent of a phase plot for \(E=1\).

An animated movie (with simplex results) is shown later below; to create the movie without the results use this code in an Rmarkdown document:

```
{r, animation.hook = 'gifski', interval = 1.5, fig.width = 9, fig.height = 5.36}
## or for portrait use fig.width = 5.36, fig.height = 8
for(iiii in 1:length(NY_lags_example$N_t)){
  plot(E_2_res,
       last.time.to.plot = iiii,
       portrait = FALSE)
}
```

A lot of information is saved in the `E_2_res` list. See `?pbsEDM::pbsEDM` for full details, but, for example, useful summary statistics are given by

```
E_2_res$results
#>   E     N_rho  N_rmse    X_rho  X_rmse
#> 1 2 0.2973217 1.12366 0.703475 1.12366
```

giving the embedding dimension \(E\) (which is 2 here because we used lags of 0 and 1), forecast skill (correlation coefficient \(\rho\)) and root-mean-square error between the observations and the predictions.

## Make predictions using different values of E

Now use the simplex algorithm for different values of embedding dimension \(E\), where, for example \(E=2\) uses first-difference values \(Y\_t\) and \(Y\_{t-1}\):

```
E_results <- pbsEDM_Evec(NY_lags_example$N_t)
```

The result is a list containing a `pbsEDM` object (as above) for each value of \(E\), the default being values \(E = 2, 3, ..., 11\):

```
summary(E_results)
#>       Length Class  Mode
#>  [1,] 26     pbsEDM list
#>  [2,] 26     pbsEDM list
#>  [3,] 26     pbsEDM list
#>  [4,] 26     pbsEDM list
#>  [5,] 26     pbsEDM list
#>  [6,] 26     pbsEDM list
#>  [7,] 26     pbsEDM list
#>  [8,] 26     pbsEDM list
#>  [9,] 26     pbsEDM list
#> [10,] 26     pbsEDM list
```

There is a plotting function for the results, showing the data (as above) plus, in the final panel, the predicted value of each data point and the \(\rho\) for each different value of \(E\):

```
plot_pbsEDM_Evec(E_results,
                 portrait = FALSE)
```

A movie of that is shown here:

**Brief technical aside for full reproducibility**. That is a saved version to avoid R package building issues. To make the movie afresh, install the `gifski` package, delete the first line in the next chunk of code, uncomment the commented line and save the movie produced here directly from the html as `pbsEDM_movie_1.gif`; see the text above the movie on this page if you want more details of this.

```
# ```{r movietime, animation.hook = 'gifski', interval = 3, fig.width = 9, fig.height = 5.36}
for(iiii in 1:length(NY_lags_example$N_t)){
  plot_pbsEDM_Evec(E_results,
       last.time.to.plot = iiii,
       portrait = FALSE)
}
```

Now see how the forecast skill (correlation coefficient \(\rho\)) varies with \(E\):

```
plot_rho_Evec(E_results)
```

Each `E_results` component corresponds to a value of \(E\), for example

```
E_2_res$results
#>   E     N_rho  N_rmse    X_rho  X_rmse
#> 1 2 0.2973217 1.12366 0.703475 1.12366
E_results[[1]]$results
#>   E     N_rho  N_rmse    X_rho  X_rmse
#> 1 2 0.2973217 1.12366 0.703475 1.12366
E_results[[2]]$results
#>   E     N_rho    N_rmse     X_rho    X_rmse
#> 1 3 0.5363073 0.8122091 0.8280765 0.8122091
E_results[[3]]$results
#>   E    N_rho    N_rmse     X_rho    X_rmse
#> 1 4 0.528308 0.8689543 0.7749746 0.8689543
```

The conclusion from the figure would be that \(E = 3\) gives the highest value of \(\rho\), and so is best used for forecasting \(\hat{N}\_{101}\). This is given by:

```
E_results[[2]]$results           # [[2]] refers to E=3
#>   E     N_rho    N_rmse     X_rho    X_rmse
#> 1 3 0.5363073 0.8122091 0.8280765 0.8122091
E_results[[2]]$Y_forecast[100]   # the forecast for Y_{100}
#> [1] -0.07706309
E_results[[2]]$N_forecast[101]   # the forecast for N_{101}
#> [1] -0.01671682
```

In this case we obtain a negative forecast of \(\hat{N}\_{101}\) (which is explored as Aspect 3).

## Movie to demonstrate the simplex algorithm for \(E=2\)

Understanding of the simplex algorithm can be enhanced with a specific animated example. With \(t^\* = 39\) we have Figure S.2 from the manuscript:

```
# ```{r EDMmovie2, animation.hook = 'gifski', interval = 8}
plot_explain_edm_movie(E_2_res,
                       tstar = 39)
```

.

Such a movie is useful for checking any single prediction. For example, with \(t^\* = 15\) (see the technical aside above to make a new movie):

```
# ```{r EDMmovie3, animation.hook = 'gifski', interval = 8}
plot_explain_edm_movie(E_2_res,
                       tstar = 15)
```

.

And changing to \(t^\* = 75\) shows the removal of an invalid candidate nearest neighbour from the library (right next to the blue solid circle) that would clearly have been used as a nearest neighbour if not removed:

```
# ```{r EDMmovie4, animation.hook = 'gifski', interval = 8}
plot_explain_edm_movie(E_2_res,
                       tstar = 75)
```

.

For the prediction of \(Y\_{100}\) we set \(t^\* = 99\) to give:

```
# ```{r EDMmovie6, animation.hook = 'gifski', interval = 8}
plot_explain_edm_movie(E_2_res,
                       tstar = 99)
```

.

In this case there are no candidate nearest neighbours to cross out, because \(t^\* = T-1\) and all defined points are candidates. This can be seen in matrix (6) in the manuscript by setting \(E=2\), \(T=100\) and \(t^\* = 99\) (besides \({\bf x}\_{t^\*}\), only rows with a \(\times\) get crossed out and since these are undefined they do not show up on the above plot anyway). Also there is no known true value of \(Y\_{100}\) in the final plot.

In the R console you can create a pdf version of any such movie with, for example:

```
plot_explain_edm_movie_save(E_2_res,
                            pdf.filename = "movie_tstar_75.pdf",
                            tstar = 75)
```

## Further details of the calculations

The intermediate calculations of the simplex algorithm are saved in the output for perusal (see `?pbsEDM`). For example, to see the nearest neighbours for each valid \(t^\*\):

```
E_2_res$neighbour_index
#>     [,1] [,2] [,3]
#> 1     NA   NA   NA
#> 2     64   61   85
#> 3     11   39   43
#> 4     12   40   44
#> 5     13   41   45
#> 6     57   88   53
#> 7     66   23   34
#> 8     67   24   95
#> 9     79   25   72
#> 10    26   46   69
#> 11     3   39   43
#> 12     4   40   44
#> 13     5   41   45
#> 14    30   73   42
#> 15    43   27   98
#> 16    28   44   82
#> 17    45   29   83
#> 18    22   61   85
#> 19    73   23   30
#> 20    24   78   82
#> 21    37   25   68
#> 22    18   61   85
#> 23    66    7   30
#> 24    67   93    8
#> 25    68    9   79
#> 26    10   46   88
#> 27    98   43   81
#> 28    16   44   82
#> 29    17   45   83
#> 30    14   73   23
#> 31    74   55   93
#> 32    91   71   56
#> 33    68   25   72
#> 34    42   38   54
#> 35    66   70    7
#> 36    67   24    8
#> 37    21   68   33
#> 38    69   54   46
#> 39    43   11   98
#> 40     4   44   12
#> 41     5   45   13
#> 42    34   38   10
#> 43    27   15   98
#> 44    16   28   82
#> 45    17   29   83
#> 46    69   26   10
#> 47    89   97   70
#> 48    52   91   71
#> 49    53   57   72
#> 50    65   85   18
#> 51    89   97   47
#> 52    48   91   56
#> 53    57   49    6
#> 54    38   65   69
#> 55    97   89   86
#> 56    91   87   52
#> 57    53   49    6
#> 58    90   62   51
#> 59    86   84   63
#> 60    87   63   80
#> 61    18   22   85
#> 62    90   58   86
#> 63    84   59   60
#> 64     2   61   60
#> 65    50   54   85
#> 66     7   23   34
#> 67     8   24   36
#> 68    33   25    9
#> 69    46   38   10
#> 70    47   89   97
#> 71    95   48   91
#> 72     9   33   49
#> 73    30   19   14
#> 74    93   78   31
#> 75    80   79   87
#> 76    80   87   49
#> 77    92   14   73
#> 78    93   24   74
#> 79     9   25   68
#> 80    60   64   87
#> 81    98   27   19
#> 82    28   20   16
#> 83    21   29   37
#> 84    63   59   60
#> 85    50   61   18
#> 86    59   62   90
#> 87    60   63   56
#> 88    26   10   46
#> 89    97   47   51
#> 90    62   58   51
#> 91    48   56   52
#> 92    77   14   30
#> 93    78   24   74
#> 94     6   57   88
#> 95    71   48    8
#> 96    94    6   57
#> 97    89   47   51
#> 98    27   81   43
#> 99    16   44   28
#> 100   NA   NA   NA
```
