# Supplementary material for: Explaining empirical dynamic modelling using verbal, graphical and mathematical approaches
Source: Ecol Evol. 2024 May 15;14(5):e10903. doi: 10.1002/ece3.10903 (PMC11094587; doi:10.1002/ece3.10903)
Supplement: Supplementary file 4 — Data S1 [file ECE3-14-e10903-s001.zip › pbsEDM-main/vignettes/aspect_2.html]

Aspect 2 demonstration


# Aspect 2 demonstration

```
library(pbsEDM)
```

## Introduction

In the manuscript, the library contains the candidate points that can be used to determine the nearest neighbours of the focal point from which we are making a prediction. Aspect 2 concerns which points can be considered as being in the library, and which should be excluded. Here we show how our suggested approach differs slightly from that in `rEDM`, giving minor changes in results. We also provide options to test alternative assumptions about which candidate neighours to exclude.


We use the simulated population time series from the manuscript, with first-differenced values denoted \(Y\_t\) at \(t = 1, 2, 3, ..., 99, 100\). We extract them from the saved tibble `NY_lags_example_3`:

```
input <-  dplyr::select(NY_lags_example_3, t, Y_t) %>%
  dplyr::rename(Time = t)
input
# A tibble: 100 × 2
    Time     Y_t
   <int>   <dbl>
 1     1 -0.0565
 2     2  0.0592
 3     3  5.24  
 4     4 -4.85  
 5     5 -0.461 
 6     6  0.273 
 7     7  0.802 
 8     8 -0.889 
 9     9 -0.203 
10    10  0.498 
# ℹ 90 more rows
```

Note that `NY_lags_example_3` already contains results. At the end of this vignette we verify that results using `rEDM` or `pbsEDM` have not changed (for example due to package updates) from those saved in `NY_lags_example_3`.

We will do the simplex calculations for embedding dimension \(E = 2\) using `rEDM` and `pbsEDM` (and also compare with some earlier independent code).

## Calculations with rEDM

First, use the `rEDM` package:

```
# library(rEDM)
packageVersion("rEDM")
[1] '1.14.0'
# We used '1.14.0' was published 7th January 2023

rEDM_res <- rEDM::Simplex(dataFrame = input,
                          columns = "Y_t",
                          target = "Y_t",
                          lib = "1 99",
                          pred = "1 99",
                          E = 2,
                          verbose = TRUE)
                          # lib and pred overlap, so leave-one-out
                          #  cross-validation is used.

rEDM_res <- rbind(c(1, input$Y_t[1], NA, NA),
                  rEDM_res)             # Add Time = 1 values, to give t = 1:100

rEDM_pred <- rEDM_res$Predictions       # Just the predicted values
```

## Calculations using pbsEDM

See the `analyse_simple_time_series` vignette for general details on using the code. Here do the simplex calculation using `pbsEDM`:

```
pbsEDM_res_full <- pbsEDM::pbsEDM(input,
                                  lags = list(Y_t = c(0:1)))   # Gives full calculations

pbsEDM_pred <- pbsEDM_res_full$X_forecast[-length(pbsEDM_res_full$X_forecast)]
                                           # Just the predictions, excluding
                                           #  t=101, so for t = 1:100
```

## Compare `rEDM` results with those from `pbsEDM`

Now to plot the `pbsEDM` predictions again those from `rEDM`, and highlight in red those that differ:

```
plot(rEDM_pred,
     pbsEDM_pred,
     xlab = "rEDM predictions",
     ylab = "pbsEDM predictions")
abline(a=0,
       b=1,
       col="grey")

# Work out the ones more than epsilon away
epsilon = 0.0001      # Small value for how different the predictions can be

different <- dplyr::tibble(t = input$Time,
                           rEDM_pred,
                           pbsEDM_pred) %>%
  dplyr::filter(abs(rEDM_pred - pbsEDM_pred) > epsilon)

points(different$rEDM_pred,
       different$pbsEDM_pred,
       col = "red",
       pch = 20)
```

The time indices correspond to the predicted times, so represent \(t^\*+1\) values, where \(t^\*\) is the focal time from which we make predictions:

```
different
# A tibble: 2 × 3
      t rEDM_pred pbsEDM_pred
  <int>     <dbl>       <dbl>
1    76     1.37        0.838
2    95     0.177       0.412
```

So there are two time indices for which results differ between `pbsEDM` and `rEDM`, corresponding to \(t^\* =\) 75 and 94.

## Explaining the differences

As defined in the manuscript, vector \(\bf{\tilde{x}}\_t\) has components defined by the lagged values of scalars \(Y\_t\). For \(E=2\) we have \[\bf{\tilde{x}}\_t = [Y\_t, ~Y\_{t-1}].\] For focal time \(t^\*\), we know \(Y\_{t^\*}\) and are trying to predict \(Y\_{t^\*+1}\).

In the manuscript we presented exclusion condition (d), namely that the library of allowable nearest neighbours cannot include any \(\bf{x}\_t\) that includes \(Y\_{t^\*+1}\), since we are trying to predict \(Y\_{t^\*+1}\).

Thus, the library does not include \[\bf{x}\_{t^\*+1} = [Y\_{t^\*+1}, ~Y\_{t^\*}]\] or \[\bf{x}\_{t^\*+2} = [Y\_{t^\*+2}, ~Y\_{t^\*+1}]\] since these both contain \(Y\_{t^\*+1}\).

### Calculations for \(t^\*=94\)

For \(t^\*=94\), we have the aforementioned discrepancy between calculations:

```
t_star <- 94
rEDM_pred[t_star + 1]
[1] 0.1773531
pbsEDM_pred[t_star + 1]
[1] 0.4123431
```

With `pbsEDM`, the nearest neighbours are saved as output, and for \(t^\*=94\) have indices and weights:

```
pbsEDM_res_full$neighbour_index[t_star,]
[1]  6 57 88
pbsEDM_res_full$neighbour_weight[t_star,]
[1] 0.3678794 0.3205861 0.2895013
```

giving the above prediction for \(Y\_{95}\) of

```
pbsEDM_pred[t_star+1]
[1] 0.4123431
```

We can verify this calculation directly from equation (13) in the manuscript using the just-calculated weights and neighbour indices:

```
sum(pbsEDM_res_full$neighbour_weight[t_star,] *
    input[pbsEDM_res_full$neighbour_index[t_star,] + 1, "Y_t"]) /
  sum(pbsEDM_res_full$neighbour_weight[t_star,])
[1] 0.4123431
```

For `rEDM` the full details of nearest neighbours do not appear to be an available output. However, here we show that \(\bf{x}\_{96}\) was used by `rEDM` as a nearest neighbour of \(\bf{x}\_{94}\). We first simply change the value of \(Y\_{95}\) so that `rEDM::Simplex()` will not pick

\[\bf{x}\_{95} = [Y\_{95},~Y\_{94}]\]

or

\[\bf{x}\_{96} = [Y\_{96}, ~Y\_{95}]\]

as a nearest neighbour of \(\bf{x}\_{94}\), and see if we get the same result as for `pbsEDM`.

```
input[t_star+1, ]
# A tibble: 1 × 2
   Time    Y_t
  <int>  <dbl>
1    95 -0.799
input_change <- input
input_change[t_star+1, "Y_t"] = 7    # Move it far out of the way so it will not be a
                                     #  nearest neighbour

# Do Simplex calculation with same options as earlier
rEDM_change_res <- rEDM::Simplex(dataFrame = input_change,
                                 columns = "Y_t",
                                 target = "Y_t",
                                 lib = "1 99",
                                 pred = "1 99",
                                 E = 2,
                                 verbose = TRUE)
rEDM_change_res <- rbind(c(1, input_change$Y_t[1], NA, NA),
                         rEDM_change_res)          # Add Time = 1 values, to give t = 1:100
rEDM_change_pred <- rEDM_change_res$Predictions    # Just the predicted values
rEDM_change_pred[t_star+1]
[1] 0.4123431
```

That is the same value as obtained earlier using `pbsEDM`.

We now do the same calculation but changing \(Y\_{96}\) so that \[\bf{x}\_{96} = [Y\_{96}, ~Y\_{95}]\] cannot be a nearest neighbour of \(\bf{x}\_{94}\) (but \(\bf{x}\_{95}\) still can be):

```
input_change <- input
input_change[t_star+2, "Y_t"] = 7    # Move it far out of the way so it will not be a
                                     #  nearest neighbour

# Do Simplex calculation with same options as earlier
rEDM_change_res <- rEDM::Simplex(dataFrame = input_change,
                                 columns = "Y_t",
                                 target = "Y_t",
                                 lib = "1 99",
                                 pred = "1 99",
                                 E = 2,
                                 verbose = TRUE)
rEDM_change_res <- rbind(c(1, input_change$Y_t[1], NA, NA),
                         rEDM_change_res)          # Add Time = 1 values, to give t = 1:100
rEDM_change_pred <- rEDM_change_res$Predictions    # Just the predicted values
rEDM_change_pred[t_star+1]
[1] 0.4123431
```

Since this again agrees with the `pbsEDM` calculation, we conclude that \[\bf{x}\_{96} = [Y\_{96}, ~Y\_{95}]\]

was being included in the library of candidate nearest neighbours by `rEDM`, since by moving \(Y\_{95}\) or \(Y\_{96}\) far away it is no longer a nearest neighbour.

As a further check, we test our new `exclusion_radius` option in `pbsEDM::pbsEDM()` with a value of 0, which should match the `rEDM` default results.

```
pbsEDM_res_excl_0_full <- pbsEDM::pbsEDM(input,
                                         lags = list(Y_t = c(0:1)),
                                         exclusion_radius = 0)
pbsEDM_pred_excl_0 <- pbsEDM_res_excl_0_full$X_forecast[-length(pbsEDM_res_excl_0_full$X_forecast)]

plot(rEDM_pred,
     pbsEDM_pred_excl_0,
     xlab = "rEDM predictions",
     ylab = "pbsEDM predictions")
abline(a=0,
       b=1,
       col="grey")
```

```
testthat::expect_equal(rEDM_pred, pbsEDM_pred_excl_0,
                       tolerance = epsilon)            # No error means they match
```

This shows that we can reproduce the `rEDM` results using `pbsEDM` with `exclusion_radius = 0`.

### Calculations for \(t^\*=75\)

We now repeat the above calculations for \(t^\*=75\). Skipping some of the explanatory steps, we have:

```
t_star <- 75
rEDM_pred[t_star + 1]
[1] 1.367744
pbsEDM_pred[t_star + 1]
[1] 0.8378772
```

as noted above (below the figure).

Using `pbsEDM` and \(t^\*=75\), the indices and weights of the nearest neighbours are:

```
pbsEDM_res_full$neighbour_index[t_star,]
[1] 80 79 87
pbsEDM_res_full$neighbour_weight[t_star,]
[1] 0.3678794 0.3227933 0.3133825
```

Change the value of \(Y\_{76}\) so that `rEDM::Simplex()` will not pick \(\bf{x}\_{76}\) or \(\bf{x}\_{77}\) as a nearest neighbour for \(\bf{x}\_{75}\):

```
input[t_star+1, ]
# A tibble: 1 × 2
   Time    Y_t
  <int>  <dbl>
1    76 -0.415
input_change <- input
input_change[t_star+1, "Y_t"] = 7    # Move it far out of the way so it will not be a
                                     #  nearest neighbour

# Do Simplex calculation with same options as earlier
rEDM_change_res <- rEDM::Simplex(dataFrame = input_change,
                                 columns = "Y_t",
                                 target = "Y_t",
                                 lib = "1 99",
                                 pred = "1 99",
                                 E = 2,
                                 verbose = TRUE)
rEDM_change_res <- rbind(c(1, input_change$Y_t[1], NA, NA),
                         rEDM_change_res)          # Add Time = 1 values, to give t = 1:100
rEDM_change_pred <- rEDM_change_res$Predictions    # Just the predicted values
rEDM_change_pred[t_star+1]
[1] 0.8378772
```

We indeed get the same result as obtained earlier using `pbsEDM`.

Now instead change \(Y\_{77}\) so that \(\bf{x}\_{77}\) cannot be a nearest neighbour (but \(\bf{x}\_{76}\) still can be):

```
input[t_star+2, ]
# A tibble: 1 × 2
   Time   Y_t
  <int> <dbl>
1    77  1.37
input_change <- input
input_change[t_star+2, "Y_t"] = 7    # Move it far out of the way so it will not be a
                                     #  nearest neighbour

# Do Simplex calculation with same options as earlier
rEDM_change_res <- rEDM::Simplex(dataFrame = input_change,
                                 columns = "Y_t",
                                 target = "Y_t",
                                 lib = "1 99",
                                 pred = "1 99",
                                 E = 2,
                                 verbose = TRUE)
rEDM_change_res <- rbind(c(1, input_change$Y_t[1], NA, NA),
                         rEDM_change_res)          # Add Time = 1 values, to give t = 1:100
rEDM_change_pred <- rEDM_change_res$Predictions    # Just the predicted values
rEDM_change_pred[t_star+1]
[1] 6.960745
```

This does not match the `pbsEDM` result. Thus, since only replacing \(Y\_{t^\*+1} = Y\_{76}\) gives the matching result to `pbsEDM`, it must be the case that `rEDM` was considering \[\bf{x}\_{t^\*+1} =
[Y\_{t^\*+1},~Y\_{t^\*}]\]

i.e.

\[\bf{x}\_{76} = [Y\_{76}, ~Y\_{75}]\] to be a nearest neighbour of \(\bf{x}\_{75}\) (and not \(\bf{x}\_{77}\)).

Note that `rEDM` prediction of 6.960745 is now very large. This is because `rEDM` is still finding \(\bf{x}\_{76}\) to be a nearest neighbour. In the next time step this moves to \(\bf{x}\_{77} = [Y\_{77}, ~Y\_{76}]\), but we have just made \(Y\_{77}\) very large and this value gets used in the calculation of \(\hat{Y}\_{76}\) (equation 13 in the manuscript), which is therefore very large.

Thus, we have demonstrated two examples for which `rEDM` has used \(Y\_{t^\* + 1}\) as a nearest neighbours (through \(\bf{x}\_{t^\*+2}\) for \(t^\*=94\) and \(\bf{x}\_{t^\*+1}\) for \(t^\*=75\)).

## Differences in \(\rho\)

Since the results differ for `pbsEDM` and `rEDM`, we expect a slight difference in the overall Pearson correlation coefficient:

```
rEDM_rho <- rEDM::ComputeError(rEDM_res$Observations,
                               rEDM_res$Predictions)$rho
rEDM_rho
[1] 0.6929147
pbsEDM_rho <- pbsEDM_res_full$results$X_rho
pbsEDM_rho
[1] 0.6950458
```

So the correlation coefficient is actually slight higher for the `pbsEDM` results compared to the `rEDM` results. Thus, for this example there is an improved fit from accommodating our suggested neighbour exclusions.

## Try alternative `exclusionRadius` settings for `rEDM`

We now try changing the `exclusionRadius` input value in `rEDM::Simplex`, which (from `?rEDM::Simplex`) “excludes vectors from the search space of nearest neighbors if their relative time index is within exclusionRadius”. This excludes temporal neighbours both forward and backwards in time, whereas we suggest (our equation (6)) to just remove the \(E\) forward neighbours.

So, re-using the above code and trying `exclusionRadius`=1 (the default is 0):

```
rEDM_res_excl_1 <- rEDM::Simplex(dataFrame = input,
                                 columns = "Y_t",
                                 target = "Y_t",
                                 lib = "1 99",
                                 pred = "1 99",
                                 E = 2,
                                 verbose = TRUE,
                                 exclusionRadius = 1)

rEDM_res_excl_1 <- rbind(c(1, input$Y_t[1], NA, NA),
                         rEDM_res_excl_1)             # Add Time = 1 values, to give t = 1:100

rEDM_pred_excl_1 <- rEDM_res_excl_1$Predictions       # Just the predicted values

different_excl <- dplyr::tibble(t = input$Time,
                                pbsEDM_pred,
                                rEDM_pred,
                                rEDM_pred_excl_1)

# See what has changed from original rEDM results
dplyr::filter(different_excl,
              abs(rEDM_pred - rEDM_pred_excl_1) > epsilon)
# A tibble: 2 × 4
      t pbsEDM_pred rEDM_pred rEDM_pred_excl_1
  <int>       <dbl>     <dbl>            <dbl>
1    76       0.838     1.37             0.838
2    77      -0.381    -0.381            1.03
```

The \(t\) values shown are for the predictions at \(t = t^\* + 1\). So the `rEDM` results have changed for \(t^\* = 75\) (which is good) and for 76 (which was not expected), but not for the desired \(t^\* = 94\).

Now check what is different to `pbsEDM` results:

```
dplyr::filter(different_excl,
              abs(pbsEDM_pred - rEDM_pred_excl_1) > epsilon)
# A tibble: 2 × 4
      t pbsEDM_pred rEDM_pred rEDM_pred_excl_1
  <int>       <dbl>     <dbl>            <dbl>
1    77      -0.381    -0.381            1.03 
2    95       0.412     0.177            0.177
```

So \(t^\* = 75\), which we just found had changed with the `exclusionRadius`=1, is not shown here meaning that it now matches the `pbsEDM` result. But \(t^\* = 94\) is still different to the `pbsEDM` results, so the correction is not global, and \(t^\* = 76\) is now different.

So we now try setting `exclusionRadius`=2:

```
rEDM_res_excl_2 <- rEDM::Simplex(dataFrame = input,
                                 columns = "Y_t",
                                 target = "Y_t",
                                 lib = "1 99",
                                 pred = "1 99",
                                 E = 2,
                                 verbose = TRUE,
                                 exclusionRadius = 2)

rEDM_res_excl_2 <- rbind(c(1, input$Y_t[1], NA, NA),
                         rEDM_res_excl_2)             # Add Time = 1 values, to give t = 1:100

rEDM_pred_excl_2 <- rEDM_res_excl_2$Predictions       # Just the predicted values

different_excl <- dplyr::mutate(different_excl,
                                rEDM_pred_excl_2)

# See what has changed from original rEDM results
dplyr::filter(different_excl,
              abs(rEDM_pred - rEDM_pred_excl_2) > epsilon)
# A tibble: 4 × 5
      t pbsEDM_pred rEDM_pred rEDM_pred_excl_1 rEDM_pred_excl_2
  <int>       <dbl>     <dbl>            <dbl>            <dbl>
1    76       0.838     1.37             0.838            0.838
2    77      -0.381    -0.381            1.03             1.03 
3    95       0.412     0.177            0.177            0.412
4    97      -0.798    -0.798           -0.798            0.755
```

So with `exclusionRadius`=2, the `rEDM` results have changed from the original `rEDM` predictions for \(t^\* = 75, 76, 94\) and \(96\). Checking what is now different to the `pbsEDM` results:

```
dplyr::filter(different_excl,
              abs(pbsEDM_pred - rEDM_pred_excl_2) > epsilon)
# A tibble: 2 × 5
      t pbsEDM_pred rEDM_pred rEDM_pred_excl_1 rEDM_pred_excl_2
  <int>       <dbl>     <dbl>            <dbl>            <dbl>
1    77      -0.381    -0.381            1.03             1.03 
2    97      -0.798    -0.798           -0.798            0.755
```

So \(t^\* = 76\) and \(96\) are different to the `pbsEDM` results. So while setting `exclusionRadius` equal to 2 seems to have led to results agreeing for \(t^\* = 75\) and \(94\), predictions now differ for \(t^\* = 76\) and \(96\).

Repeating the above with `exclusionRadius`=3 leads to five differences with the `pbsEDM` results (results not shown).

So, as expected, changing `exclusionRadius` in the call to `rEDM::Simplex()` does not give exactly the same results as our suggested default.

## Check our `exclusion_radius` setting

We also try `exclusion_radius = 7` in `pbsEDM::pbsEDM()`, just picking 7 as a new example, as a further check of our code, comparing with `rEDM` calculations:

```
pbsEDM_res_excl_7_full <- pbsEDM::pbsEDM(input,
                                         lags = list(Y_t = c(0:1)),
                                         exclusion_radius = 7)
pbsEDM_pred_excl_7 <- pbsEDM_res_excl_7_full$X_forecast[-length(pbsEDM_res_excl_7_full$X_forecast)]

rEDM_res_7 <- rEDM::Simplex(dataFrame = input,
                            columns = "Y_t",
                            target = "Y_t",
                            lib = "1 99",
                            pred = "1 99",
                            E = 2,
                            verbose = TRUE,
                            exclusionRadius = 7)

rEDM_res_7 <- rbind(c(1, input$Y_t[1], NA, NA),
                  rEDM_res_7)             # Add Time = 1 values, to give t = 1:100

rEDM_pred_7 <- rEDM_res_7$Predictions       # Just the predicted values

testthat::expect_equal(rEDM_pred_7, pbsEDM_pred_excl_7,
                       tolerance = epsilon)            # No error means they match
```

Thus, the calculations agree (and we checke they also do for all values of `exclusion_radius` up to 10).

## Independent code

Some of the above differences were originally figured out using other independent code (written by Andrew Edwards) to help understand EDM. The results are saved as `NY_lags_example_3$my.pred`. The predictions agree with those calculated here using `pbsEDM`:

```
testthat::expect_equal(NY_lags_example_3$my.pred,
                       pbsEDM_pred)
```

The original code also allowed independent verification of the above results (for example by explicitly allowing \(Y\_{t^\* + 1}\) to be used as a nearest neighbour). The original code is available on request, but is not functionalised or generalisable for \(E>2\), and is now superceded by the functions in `pbsEDM`. Note that writing of the `pbsEDM` code was led by Luke Rogers, using functions that were independently written from the original code. Hence the original code also served as an independent check of some of the `pbsEDM` code.

## Test that rEDM and pbsEDM results have not changed

Here we run some tests to check that if any of the above results from `pbsEDM` or `rEDM` have changed from when this vignette was written (some results are saved in `NY_lags_example_3`). The tests will give errors if they fail (and any such failures should be investigated and this vignette updated).

Check the default `rEDM` predictions have not changed.

```
testthat::expect_equal(NY_lags_example_3$rEDM.pred,
                       rEDM_res$Predictions)       # If no error then they match
```

Also check that the results with different values of `exclusionsRadius` have not changed:

```
testthat::expect_equal(check_rEDM_excl_1, rEDM_pred_excl_1)
testthat::expect_equal(check_rEDM_excl_2, rEDM_pred_excl_2)
```

The test in the `Independent code` section has already verified that the `pbsEDM` results have not changed (since they match the `my.pred` results from the original independent code).
