# Supplementary material for: Explaining empirical dynamic modelling using verbal, graphical and mathematical approaches
Source: Ecol Evol. 2024 May 15;14(5):e10903. doi: 10.1002/ece3.10903 (PMC11094587; doi:10.1002/ece3.10903)
Supplement: Supplementary file 4 — Data S1 [file ECE3-14-e10903-s001.zip › pbsEDM-main/vignettes/negative_predictions.html]

Negative predictions and differences in correlation coefficients


# Negative predictions and differences in correlation coefficients

```
library(pbsEDM)
```

## Introduction

Here we provide reproducible details of our results in the manuscript that predicted values of lagged variables can give unrealistic negative predictions of the original unlagged populations, and demonstrate a simple proposed solution. We also show how correlations based on predictions of lagged variables can differ somewhat from those based on the predictions of the actual populations (which is what we are interested in).

Again we use the simulated population time series from the manuscript, with population size denoted \(N\_t\) and first-differenced values denoted \(Y\_t\) at \(t = 1, 2, 3, ..., 99, 100\). We extract them from the saved tibble `NY_lags_example_3`:

```
input <-  dplyr::select(NY_lags_example_3, t, N_t, Y_t) %>%
  dplyr::rename(Time = t)
input
# A tibble: 100 × 3
    Time    N_t     Y_t
   <int>  <dbl>   <dbl>
 1     1 0.0995 -0.0565
 2     2 0.0430  0.0592
 3     3 0.102   5.24  
 4     4 5.34   -4.85  
 5     5 0.489  -0.461 
 6     6 0.0281  0.273 
 7     7 0.301   0.802 
 8     8 1.10   -0.889 
 9     9 0.214  -0.203 
10    10 0.0116  0.498 
# ℹ 90 more rows
```

## Calculations using pbsEDM (as done in the aspect 2 vignette)

See the `analyse_simple_time_series` vignette for general details on using the code. Here do the simplex calculation for \(E = 2\):

```
pbsEDM_res_full <- pbsEDM::pbsEDM(input,
                                  lags = list(Y_t = c(0:1)))   # Gives full calculations

pbsEDM_pred_Y <- pbsEDM_res_full$X_forecast[-length(pbsEDM_res_full$X_forecast)]
                                           # Just the predictions, excluding
                                           #  t=101, so for t = 1:100
```

## Explaining how negative population predictions arise

Use the \(Y\_t\) calculations and then convert to \(N\_t\). Equation (1) in the manuscript defines \(Y\_t\) as:

\(Y\_t = N\_{t+1} - N\_t\).

So having estimated \(\hat{Y}\_t\) we can rearrange that to estimate \(\hat{N}\_{t+1}\) as:

\(\hat{N}\_{t+1} = N\_t + \hat{Y}\_t\)

or

\(\hat{N}\_t = N\_{t-1} + \hat{Y}\_{t-1}\),

to give a vector of estimated \(\hat{N}\_t\) values:

```
pbsEDM_pred_N = c(NA,
                  input$N_t + pbsEDM_pred_Y)
pbsEDM_pred_N
  [1]           NA           NA           NA  0.289056951  0.080767036
  [6]  0.323171929  0.928236616  0.564487644  0.160840312  0.214919277
 [11]  0.188081305  1.870277859  0.848967821 -0.186782556  0.472983601
 [16]  1.511592770  0.264533104  0.129067595  0.537826197  1.312944032
 [21]  1.189225618  0.035381865  0.492890733  1.724009413  0.596981270
 [26]  0.292465039  0.336031436  3.270535429 -0.209085029 -0.043220123
 [31]  0.246312809  1.629919507  0.864091894  0.524613472  0.654938572
 [36]  3.127322908  0.569965314 -0.004757061  0.572585015  0.811802618
 [41]  0.542189068 -0.099017870  0.595349716  2.449084662  0.463668377
 [46]  0.102119992  0.340738472  2.556576048  1.754532592  0.589200849
 [51]  0.670929400  0.943198372  1.150053582  0.455634536  0.458084504
 [56]  2.777014513  1.260254677  0.680811701  0.673512249 -0.089713336
 [61]  0.161031341  0.884064826  1.201644648 -0.327110872  0.354760473
 [66]  3.091878781  0.648183475  0.533000180  0.162013062  0.838731150
 [71]  2.721361096  1.500177086  0.656452630  0.729903148  1.571699704
 [76]  0.868052867  1.432177984 -0.202367304  1.722143019  0.698477857
 [81]  0.862226246  0.525000396 -0.273715469  0.172071468  0.963282419
 [86]  0.256963514  1.069679229  0.191447880  0.234257651  3.292525084
 [91]  2.932298485  0.672288601  0.197920085  1.443603711  0.203009936
 [96]  1.404749959  0.155923957 -0.164964599  0.889735172  0.593538416
[101] -0.075774284
```

The first three `NA` values demonstrate that we cannot predict \(\hat{N}\_1\) (because we do not know \(N\_0\)), or \(\hat{N}\_2\) and \(\hat{N}\_3\) (because we cannot predict \(\hat{Y}\_1\) or \(\hat{Y}\_2\)).

Of note are the several negative predictions of \(\hat{N}\_t\), which are unrealistic because our data represent populations (and negative zooplankton, for example, do not exist. The indices of these give the predicted time values (i.e. \(t^\* + 1\)):

```
which(pbsEDM_pred_N < 0)
 [1]  14  29  30  38  42  60  64  78  83  98 101
```

Of particular interest is the negative prediction for \(\hat{N}\_{101}\) of -0.0757743, since the aim of the analysis is generally to forecast next year’s population from all currently available data.

## Simple solution

The simplest solution this is to just replace the negative predictions \(\hat{N}\_t\) with the minimum observed value of \(N\_t\).

```
N_t_min <- min(input$N_t)

pbsEDM_pred_N_replace <- pbsEDM_pred_N
pbsEDM_pred_N_replace[pbsEDM_pred_N_replace < 0] = N_t_min
```

Graphically this is shown by plotting all the original \((N\_t, \hat{N}\_t)\) points as open black circles, then the set with the negative \(\hat{N}\_t\) values replaced as red filled circles (so that 11 points can be seen to change; the 1:1 line is added in black):

```
plot(c(input$N_t, NA),
     pbsEDM_pred_N,
     xlab = "Observed N_t",
     ylab = "Predicted N_t",
     main = "Red: change -ve predictions to min +ve observation")

points(c(input$N_t, NA),
       pbsEDM_pred_N_replace,
       pch = 20,
       col = "red")
abline(a = 0, b = 1)
```

```
# abline(h = mean_pred_N)
# abline(h = mean_pred_N_replace,
#        col = "red")
# abline(v = mean(input$N_t),
#        col = "grey")

# lm_fit  <- lm(pbsEDM_pred_N ~ c(input$N_t, NA))
# abline(lm_fit)
# lm_fit_replace  <- lm(pbsEDM_pred_N_replace ~ c(input$N_t, NA))
# abline(lm_fit_replace, col = "red")
```

## A good fit for the first-differenced values (\(Y\_t\)) does not necessarily translate to a good fit to the original population sizes (\(N\_t\))

The above simple fix removes unrealistic negative predictions. However, the figure raises a fundamental issue, in that the predicted values \(\hat{N}\_t\) do not seem to match the original data \(N\_t\) too well.

The correlation coefficient of \(\hat{N}\_t\) and \(N\_t\) before correcting the negative values is:

```
pbsEDM_rho_N <- cor(c(input$N_t, NA),
                    pbsEDM_pred_N,
                    use = "pairwise.complete.obs")
pbsEDM_rho_N
[1] 0.2816586
```

and after correcting the negative values it is

```
pbsEDM_rho_N_replace <- cor(c(input$N_t, NA),
                            pbsEDM_pred_N_replace,
                            use = "pairwise.complete.obs")
pbsEDM_rho_N_replace    # cor(input$N_t[4:100], pbsEDM_pred_N_replace[4:100]) gives same
[1] 0.2815723
```

The latter \(\rho\) with the negatives corrected is (unintuitively) very slightly lower than the original value. But the more important point is that a \(\rho\) of 0.28 is much lower than that based on \(Y\_t\) and \(\hat{Y}\_t\), which is

```
pbsEDM_rho_Y <- pbsEDM_res_full$results$X_rho
pbsEDM_rho_Y
[1] 0.6950458
```

We highlight it as something for practioners to be aware of.

Plotting \(\hat{Y}\_t\) against \(Y\_t\) (these are the \(E=2\) points in the manuscript’s Figure~1(f):

```
plot(input$Y_t,
     pbsEDM_pred_Y,
     xlab = "Observed Y",
     ylab = "Predicted Y")
abline(a=0, b=1)
```

shows a good correspondence, as given by the correlation coefficient of 0.6950458.

Thus, a good correspondence based on the first-differenced \(Y\_t\) values does not necessarily lead to good correspondence in the \(N\_t\) values, which is what we are interested in for practical situations. Hence our recommendation that practitioners also check the fits of the predictions based on non-first-differenced values (\(N\_t\)) and not just first-differenced values (\(Y\_t\)).

## Repeating analyses with \(E=3\)

The above was all for the simplest embedding dimension of \(E=2\). We repeat the calculations here for \(E=3\), which is the optimal embedding dimension (based on the maximum \(\rho\) value), and so should be used for forecasting. The first-differencing calculations remain the same, the higher embedding dimension just allows more lags (and more neighbours) to be used in the making predictions.

```
pbsEDM_res_full_E_3 <- pbsEDM::pbsEDM(input,
                                      lags = list(Y_t = c(0:2)))   # Gives full calculations

pbsEDM_pred_Y_E_3 <- pbsEDM_res_full_E_3$X_forecast[-length(pbsEDM_res_full$X_forecast)]
                                           # Just the predictions, excluding
                                           #  t=101, so for t = 1:100
```

```
pbsEDM_pred_N_E_3 = c(NA,
                  input$N_t + pbsEDM_pred_Y_E_3)
pbsEDM_pred_N_E_3
  [1]          NA          NA          NA          NA  0.10834726  0.33008215
  [7]  1.04036231  2.15592299  0.37484762  0.09915660  0.60033270  1.59956925
 [13]  0.87171962 -0.15335391  0.40039725  2.50894451  0.49583773  0.10635031
 [19]  0.69242754  0.47099554  0.18043911  0.10409712  0.18602403  2.21020567
 [25]  0.13268314  0.10812465  0.36672820  2.64714822  0.13385234 -0.04919500
 [31]  0.23476617  2.42319229  0.69172740 -0.03731750  0.64090651  3.44270949
 [37]  0.59600482  0.02323667  0.71649997  1.67828584  0.38803656 -0.08031999
 [43]  0.58042342  1.88830299  0.56424862  0.10137357  0.61261156  1.86142929
 [49]  0.33742196  0.38147960  0.71561119  1.22174880  0.09897486  0.52585366
 [55]  0.52999263  0.69456827  0.20316213  0.41659986  0.45044799  0.41317003
 [61]  0.14350786  0.32691113  0.54000740 -0.05256441  0.24630492  0.25841100
 [67]  0.41682371  0.33920542  0.15695968  0.79403825  3.52931316  0.67379832
 [73]  0.43361838  0.57137651  0.93463812  0.56336821  0.73657075  1.01210337
 [79]  0.42935157  0.71637109  0.60004653  1.13442686  0.28179738  0.13252378
 [85]  0.95989989  1.93591645  0.81300598  0.15110446  0.22055427  0.79121191
 [91]  0.18114840  0.72204494  0.27225152  0.89213949  0.12320664  1.31670810
 [97]  0.67552555  1.07061602  0.83889444  0.68944671 -0.01671682
```

Interestingly, there are fewer negative predictions of \(\hat{N}\_t\) for \(E=3\) than the 11 we had for \(E=2\). The indices of these give the predicted time values (i.e. \(t^\* + 1\)):

```
which(pbsEDM_pred_N_E_3 < 0)
[1]  14  30  34  42  64 101
```

Though there is still a negative prediction for \(\hat{N}\_{101}\) of -0.0167168.

Now replace the negative predictions \(\hat{N}\_t\) with the minimum observed value of \(N\_t\).

```
N_t_min <- min(input$N_t)

pbsEDM_pred_N_E_3_replace <- pbsEDM_pred_N_E_3
pbsEDM_pred_N_E_3_replace[pbsEDM_pred_N_E_3_replace < 0] = N_t_min
```

And repeat the above figure:

The above figure does look better (close to the 1:1 line) than for \(E=2\). The correlation coefficient of \(\hat{N}\_t\) and \(N\_t\) before correcting the negative values is:

```
pbsEDM_rho_N_E_3 <- cor(c(input$N_t, NA),
                        pbsEDM_pred_N_E_3,
                        use = "pairwise.complete.obs")
pbsEDM_rho_N_E_3
[1] 0.5367339
```

and after correcting the negative values it is

```
pbsEDM_rho_N_E_3_replace <- cor(c(input$N_t, NA),
                                pbsEDM_pred_N_E_3_replace,
                                use = "pairwise.complete.obs")
pbsEDM_rho_N_E_3_replace
[1] 0.5361547
```

which again is slightly lower than the original value. But the \(\rho\) has improved somewhat from that for \(E=2\), but ist still not as high as based on \(Y\_t\) and \(\hat{Y}\_t\), which is

```
pbsEDM_rho_Y_E_3 <- pbsEDM_res_full_E_3$results$X_rho
pbsEDM_rho_Y_E_3
[1] 0.8281812
```

As above, plotting \(\hat{Y}\_t\) against \(Y\_t\) (these are the \(E=3\) points in the manuscript’s Figure~1(f):

```
plot(input$Y_t,
     pbsEDM_pred_Y_E_3,
     xlab = "Observed Y",
     ylab = "Predicted Y")
abline(a=0, b=1)
```

shows a good correspondence, as given by the correlation coefficient of 0.8281812.

## Test that the results have not changed

Here we run some tests to check that if any of the above results from `pbsEDM` have changed from when this vignette was written (some results are saved in `NY_lags_example_3`). The tests will give errors if they fail (and any such failures should be investigated and this vignette updated).

Check the `pbsEDM` \(Y\_t\) predictions for \(E=2\) have not changed:

```
testthat::expect_equal(NY_lags_example_3$my.pred,
                       pbsEDM_pred_Y)             # If no error then they still match
```
