# Supplementary material for: Explaining empirical dynamic modelling using verbal, graphical and mathematical approaches
Source: Ecol Evol. 2024 May 15;14(5):e10903. doi: 10.1002/ece3.10903 (PMC11094587; doi:10.1002/ece3.10903)
Supplement: Supplementary file 4 — Data S1 [file ECE3-14-e10903-s001.zip › pbsEDM-main/vignettes/pbsSmap.html]

pbsSmap example


# pbsSmap example

```
library(pbsEDM)
```

## Introduction

Example use of the `pbsSmap()` function for implementing the S-map algorithm, giving the results reported in the manuscript.

Again we use the simulated population time series from the manuscript, with population size denoted \(N\_t\) and first-differenced values denoted \(Y\_t\) at \(t = 1, 2, 3, ..., 99, 100\). We extract them from the saved tibble `NY_lags_example_3`:

```
input <-  dplyr::select(NY_lags_example_3, t, N_t, Y_t) %>%
  dplyr::rename(Time = t)
input
# A tibble: 100 × 3
    Time    N_t     Y_t
   <int>  <dbl>   <dbl>
 1     1 0.0995 -0.0565
 2     2 0.0430  0.0592
 3     3 0.102   5.24  
 4     4 5.34   -4.85  
 5     5 0.489  -0.461 
 6     6 0.0281  0.273 
 7     7 0.301   0.802 
 8     8 1.10   -0.889 
 9     9 0.214  -0.203 
10    10 0.0116  0.498 
# ℹ 90 more rows
```

In the `negative_predictions` vignette we used the simplex algorithm and found the optimal embedding dimension to be \(E = 3\), so we use that here.

```
E_fix <- 3
```

## Calculations using pbsEDM

Since \(E = 3\) we use lags of 0, 1, and 2, and test over a range of \(\theta\), where \(\theta\) represents the degree of local weighting:

```
theta_vec = seq(0, 5, 0.1)
rho_vec <- smap_thetavec(input,
                         lags = list(Y_t = 0:2),
                         theta_vec = theta_vec)

plot(theta_vec,
     rho_vec,
     xlab = expression("Degree of local weighting, " * theta),
     ylab = expression("Forecast skill, " * rho),
     ylim = c(0.7, 1))
```

Thus we can see that the forecast skill (measured by the correlation coefficient \(\rho\)) increases as \(\theta\) increases. The value \(\theta = 0\) represents the global linear solution, with the solutions becoming more locally weighted (and hence nonlinear) as \(\theta\) increases.

The maximum value of \(\rho\) is, with corresponding \(\theta\):

```
max(rho_vec)
[1] 0.8675023
theta_max <- theta_vec[which.max(rho_vec)]
theta_max
[1] 2.7
```

We then test if this is signficantly different to the linear system with \(\theta = 0\):

```
rho_vec[1]     # rho at theta=0
[1] 0.8141303
p_val <- smap_surrogates(input,
                         lags = list(Y_t = 0:2),
                         theta = theta_max)
p_val
[1] 0.29
```

Since the p-value is \(>0.05\), we conclude that there is not sufficient evidence that the system is nonlinear, since the fit is not significantly better to that from using \(\theta = 0\). However, the focus of our application is in making projections, so we use the optimal \(\theta\) for that:

```
Smap_optimal <- pbsSmap(input,
                        lags = list(Y_t = 0:2),
                        theta = theta_max)
pbsEDM: 5.68 sec elapsed

Yhat_100 <- Smap_optimal$Y_forecast[100]
Yhat_100
[1] 0.4801684
Nhat_101 <- Yhat_100 + input$N_t[100]
Nhat_101
[1] 0.5405147
```

The latter gives \(\hat{N}\_{101}\), the forecast of the population at time step 101. This is different to the -0.017 calculated using just the simplex algorithm with \(E=3\) in the `negative_predictions` vignette. So prediction of a negative future population is avoided here, but there is no guarantee of this with the S-map algorithm.

Related to this, we can check how many negative projected values (across the whole time series) arise from S-map calculations:

```
pbsSmap_pred_Y <- Smap_optimal$Y_forecast[-length(Smap_optimal$Y_forecast)]
                                           # Just the predictions for Y_t, excluding
                                           #  t=101, so for t = 1:100
pbsSmap_pred_N = c(NA,
                   input$N_t + pbsSmap_pred_Y)
pbsSmap_pred_N
  [1]           NA           NA           NA           NA  0.103841240
  [6]  0.079125650  0.739370954  3.142444151  0.685989840  0.064050236
 [11]  0.409693475  1.534270970  0.455187736 -0.024565077  0.650119297
 [16]  2.653714835  0.245015508  0.069707992  1.232398298  1.130559534
 [21]  0.122915005  0.020704545  0.311005294  2.132515837  0.182068387
 [26]  0.031733169  0.317484456  2.320414797  0.212666294 -0.001226816
 [31]  0.184861873  1.845221770  0.175373760  0.102559879  1.614172993
 [36]  2.050564426  0.007597273  0.079158918  0.966481705  1.806348257
 [41]  0.196919158  0.101828711  0.731637504  1.791430661  0.052147343
 [46]  0.037245171  0.688286846  1.866914670  0.275145398  0.098756112
 [51]  0.628003849  1.327268872  0.106881395  0.124136149  0.427635856
 [56]  0.988927542  0.083073072  0.231856071  0.808777419  1.035261411
 [61]  0.011387284  0.164890929  0.453452926  0.299313154  0.068079976
 [66]  0.275073015  0.385541608  0.069340084  0.225635219  0.653745995
 [71]  2.586563703  0.384626401  0.295163746  0.707595665  1.462241767
 [76]  0.215499768  0.260351229  1.824435565  1.330459570  0.732877034
 [81]  0.238768309  1.815685392  0.608031068  0.377594187  0.395814113
 [86]  1.714463574  0.306720796  0.368145652  0.284513353  0.655274469
 [91]  0.598268650  0.050324811  0.544790576  0.740711432  0.894062317
 [96]  0.147421598  1.394991491  0.595663169  1.250553350 -0.056340803
[101]  0.540514661
which(pbsSmap_pred_N < 0)
[1]  14  30 100
```

Thus there are only three time indices for which we obtain negative predictions of \(N\_t\) when using S-map, compared to six when using the simplex algorithm. This difference may be due to the higher correlation coefficient comparing predictions of \(Y\_t\) to actual values when using S-map compared to using simplex.

## Calculations using rEDM

We now do the same S-map calculations using code from the `rEDM` package, to see how the library of candidate neighbours compares to our default `pbsEDM` library.

```
rEDM_Smap <- rEDM::PredictNonlinear(dataFrame = input,
                                    columns = "Y_t",
                                    target = "Y_t",
                                    lib = "1 99",
                                    pred = "1 99",
                                    theta = theta_vec,
                                    E = 3,
                                    verbose = TRUE,
                                    showPlot = FALSE)
plot(theta_vec,
     rho_vec,
     xlab = expression("Degree of local weighting, " * theta),
     ylab = expression("Forecast skill, " * rho),
     ylim = c(0.7, 1))
lines(theta_vec,
      rEDM_Smap$rho,
      col = "red")
```

The red line gives the `rEDM` results, which are close, but not exactly the same, as those from `pbsEDM` (black circles) derived earlier. We now use `rEDM::SMap()` for the previously calculated `theta_max`, as it gives more detailed output:

```
rEDM_Smap_full <- rEDM::SMap(dataFrame = input,
                             columns = "Y_t",
                             target = "Y_t",
                             lib = "1 99",
                             pred = "1 99",
                             theta = theta_max,
                             E = 3,
                             verbose = TRUE)
rEDM_Smap_pred_Y <- c(NA,
                      NA,
                      rEDM_Smap_full$predictions$Predictions)

plot(rEDM_Smap_pred_Y,
     pbsSmap_pred_Y,
     xlab = expression("rEDM predictions of " * Y_t),
     ylab = expression("pbsSmap predictions of " * Y_t))
abline(0, 1)
```

So the predicted values from the two sets of code are certainly similar, but not exactly the same.

Work out which ones appear most different:

```
epsilon <- 0.14     # Gives the four most different
different <- dplyr::tibble(t = input$Time,
                           rEDM_Smap_pred_Y,
                           pbsSmap_pred_Y) %>%
  dplyr::mutate(diff = rEDM_Smap_pred_Y - pbsSmap_pred_Y)

different_few <- dplyr::filter(different,
                               abs(diff) > epsilon)

different_few
# A tibble: 4 × 4
      t rEDM_Smap_pred_Y pbsSmap_pred_Y   diff
  <int>            <dbl>          <dbl>  <dbl>
1    15            1.35          1.52   -0.172
2    31            0.428         0.672  -0.245
3    61            0.225         0.0775  0.147
4    94            0.191         0.384  -0.193

plot(rEDM_Smap_pred_Y,
     pbsSmap_pred_Y,
     xlab = expression("rEDM predictions of " * Y_t),
     ylab = expression("pbsSmap predictions of " * Y_t))
abline(0, 1)

points(different_few$rEDM_Smap_pred_Y,
       different_few$pbsSmap_pred_Y,
       col = "red",
       pch = 20)
```

So the largest difference occurs for \(Y\_{31}\), for which \(t^\* = 30\).

The `pbsEDM` output contains full details of calculations, so we can look at the neighbours of \(\bf{x}\_{30}\) (with the closest given first):

```
t_star <- 30
Smap_optimal$neighbour_index[t_star, ]
  [1] 46 18 38 84 22 34 69 26 42 73 10 97 50 80 77  7 54 58 23 19 81 89  6 65 27
 [26] 88 76 66 70 47 61 62 95 35 92 51 15 57 86 85 14 64 43 55 93 59 53 63 60 49
 [51] 87 98 90 96 72 78 91 52 74 56 48  8 71 39  9 24 37 67 68 94 25 20 36 75 79
 [76] 82 21 11  3 83 28 29 17 16 44 45 40 41  4 12  5 13 NA NA NA NA NA NA NA NA
[101] NA
```

By sorting them we can see the indices in the library:

```
sort(Smap_optimal$neighbour_index[t_star, ])
 [1]  3  4  5  6  7  8  9 10 11 12 13 14 15 16 17 18 19 20 21 22 23 24 25 26 27
[26] 28 29 34 35 36 37 38 39 40 41 42 43 44 45 46 47 48 49 50 51 52 53 54 55 56
[51] 57 58 59 60 61 62 63 64 65 66 67 68 69 70 71 72 73 74 75 76 77 78 79 80 81
[76] 82 83 84 85 86 87 88 89 90 91 92 93 94 95 96 97 98
```

This matches equation (7) in the manuscript, with the omitted indices in equation (6) given by

```
c(1:(E_fix - 1),
  setdiff(E_fix:nrow(input),
          sort(Smap_optimal$neighbour_index[t_star, ])))
[1]   1   2  30  31  32  33  99 100
```

We wish to see whether some of those indices are included in the library of candidate neighbours for the S-map calculations in `rEDM`, but detailed output regarding nearest neighbours etc. is not available from the `rEDM` calculations.

So first we visualise the focal point \({\bf x}\_{t^\*}\) and the three subsequent points that should be omitted from the library (as per equations 6 or 7), namely \({\bf x}\_{t^\*+1}\), \({\bf x}\_{t^\*+2}\), and \({\bf x}\_{t^\*+3}\). We can use the `pbsEDM::plot_phase_3d()` function (which requires an input of class `pbsEDM` so we do the calculation here); for \(t^\* = 30\):

```
pbsEDM_simplex <- pbsEDM(NY_lags_example,
                         lags = list(Y_t = 0:2))
plot_phase_3d(pbsEDM_simplex,
              tstar = 30,
              early.col.lines = NA)
```

The blue dot is \({\bf x}\_{30}\), and the red dots are \({\bf x}\_{31}\), \({\bf x}\_{32}\), and \({\bf x}\_{33}\). These are not the nearest neighbours in this case, but note that their inclusion would still affect the mean distance of all points from the focal point (equation S.14 in the manuscript), and hence affect the estimate of \({\bf x}\_{31}\).

However, for \(t^\*=93\) (the value with the second largest different between the `pbsEDM` and `rEDM` results) we have:

```
plot_phase_3d(pbsEDM_simplex,
              tstar = 93,
              early.col.lines = NA)
```

for which one of the red points is very close to the blue \({\bf x}\_{93}\), and so its inclusion in the library would surely affect the prediction of \({\hat Y}\_{94}\).

We can test that assertion by including the extra three points as later dummy data and re-running the analysis in `pbsEDM`; being later in the time series they will not get automatically excluded because they will not be close in time to \(t^\*\):

```
t_star = 93
pbsEDM_simplex$X[t_star:(t_star+3), ]    # x(tstar) and next three
          Y_t_0      Y_t_1      Y_t_2
[1,] -0.7916655  1.1466821 -0.6579462
[2,]  0.4825339 -0.7916655  1.1466821
[3,] -0.7987376  0.4825339 -0.7916655
[4,]  0.4390243 -0.7987376  0.4825339

# It looks like the third one here is the closest to the the first row, verified
#  by the 0.677 value in:
dist(pbsEDM_simplex$X[t_star:(t_star+3), ])
          1         2         3
2 2.9389554                    
3 0.6775129 2.6499872          
4 2.5690367 0.6656095 2.1902728

# These are the distances of the four nearest neighbours from the simplex calc:
pbsEDM_simplex$neighbour_distance[t_star, ]
[1] 0.3430359 0.7338678 0.9415294 0.9579622

# So x(tstar+2) would have been the second nearest neighbour, but it is
#  excluded here from the simplex calculation.
# So now add the necessary values as dummy data, re-run S-map from pbsSmap, and
#  see if it gets close to the rEDM calculation:

input_dummy <- rbind(input[1:99, ],
                     input[(t_star - 1):(t_star + 3), ],
                     input[100, ])

Smap_optimal_dummy <- pbsSmap(input_dummy,
                              lags = list(Y_t = 0:2),
                              theta = theta_max)
pbsEDM: 6.22 sec elapsed

Smap_optimal_dummy$Y_forecast[t_star+1]
[1] 0.1940898
```

That estimate is very close to that from `rEDM`:

```
rEDM_Smap_pred_Y[t_star+1]
[1] 0.1914508
```

and somewhat different to the original `pbsEDM` estimate:

```
Smap_optimal$Y_forecast[t_star+1]
[1] 0.3841894
```

The actual nearest neighbours are

```
Smap_optimal_dummy$neighbour_index[t_star, ]
  [1]  78 103  74  24  31   8  67  97  20  55  89  86  47  71  91  52  62  58
 [19]  59  48  51  70  63  36  82  80  87  50  60  56  76  65  64  54  90  35
 [37]  61  85  69   7  84  10  38  26  22  34  66  88  57  53  49  23  32 101
 [55]  28  72  33  73  19  99   9  75  77  92  68  15  18  16  25  79  44 102
 [73]  30  37  46  27  81  43  98  21  42  83  40  39   6  29  17  45  14 100
 [91]   4  11  12   3  41   5  13  NA  NA  NA  NA  NA  NA  NA  NA  NA
```

So the second nearest neighbour now is indeed for \(t=103\), which is the dummy point in the state space added in, corresponding to \(t=95\). So \(t=95\) was originally correctly excluded from the library of neighbours in `pbsSmap`, but by adding it as dummy data later we allow that neighbour to be included as \(t=103\). The result is close to that from `rEDM`, suggesting that \(t=95\) is included in the library for \(t^\* = 93\), unlike in `pbsSmap`.

Note that the estimates of \(\hat{Y}\_{94}\) from `rEDM` and from `pbsEDM` with the dummy data added do not match exactly because the dummy \(Y\_t\) values create extra \({\bf x}\_t\) points, which affect the mean of the distances of all points from \({\bf x}\_{93}\).

## Test that pbsEDM and rEDM results have not changed

Just check that the results have not changed (due to any updates in the packages)::

```
testthat::expect_equal(Nhat_101,
                       0.5405147,
                       tolerance = 0.000001)       # If no errors then they match
testthat::expect_equal(different_few$diff,
                       c(-0.1724516, -0.2447201,  0.1473963, -0.1927386),
                       tolerance = 0.000001)
```
