# Supplementary figures and images for: Explaining empirical dynamic modelling using verbal, graphical and mathematical approaches
Source: Ecol Evol. 2024 May 15;14(5):e10903. doi: 10.1002/ece3.10903 (PMC11094587; doi:10.1002/ece3.10903)

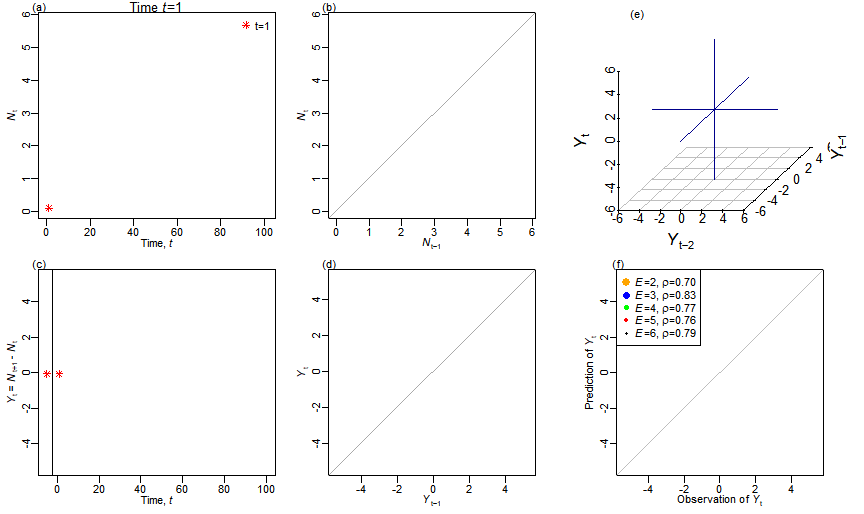

Supplement: Supplementary file 4 — Data S1 [file ECE3-14-e10903-s001.zip › pbsEDM-main/vignettes/pbsEDM_movie_1.gif]

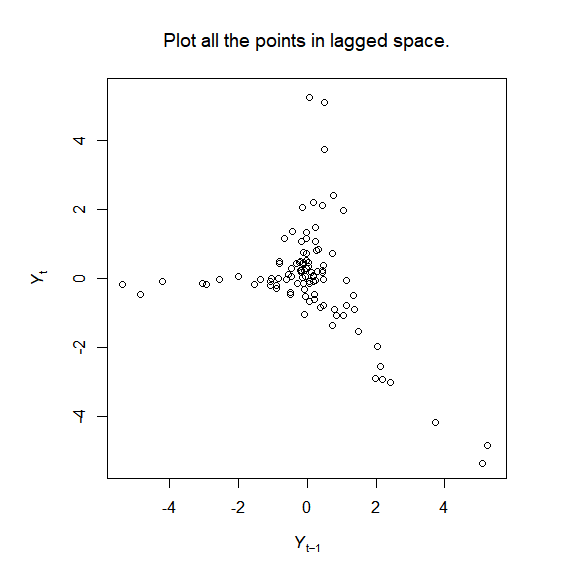

Supplement: Supplementary file 4 — Data S1 [file ECE3-14-e10903-s001.zip › pbsEDM-main/vignettes/pbsEDM_movie_tstar_15.gif]

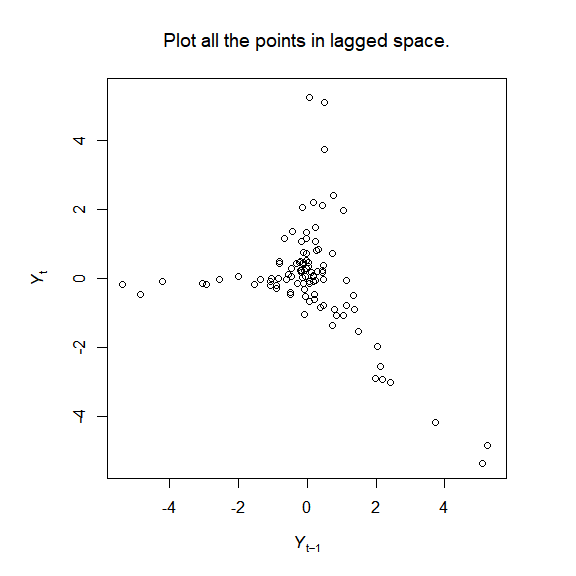

Supplement: Supplementary file 4 — Data S1 [file ECE3-14-e10903-s001.zip › pbsEDM-main/vignettes/pbsEDM_movie_tstar_39.gif]

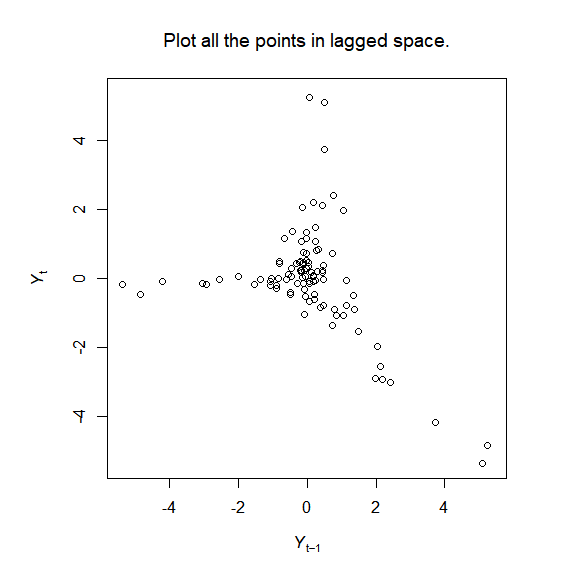

Supplement: Supplementary file 4 — Data S1 [file ECE3-14-e10903-s001.zip › pbsEDM-main/vignettes/pbsEDM_movie_tstar_75.gif]

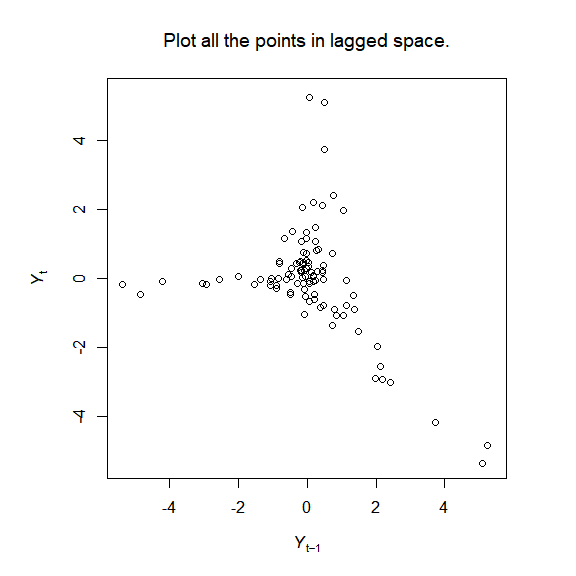

Supplement: Supplementary file 4 — Data S1 [file ECE3-14-e10903-s001.zip › pbsEDM-main/vignettes/pbsEDM_movie_tstar_99.gif]
